# Phylogeny and biogeography of Indochinese freshwater mussels in the genus *Pilsbryoconcha* Simpson, 1900 (Bivalvia: Unionidae) with descriptions of four new species

**DOI:** 10.1038/s41598-022-24844-9

**Published:** 2022-11-28

**Authors:** Ekgachai Jeratthitikul, Siwanon Paphatmethin, Chirasak Sutcharit, Peng Bun Ngor, Khamla Inkhavilay, Pongpun Prasankok

**Affiliations:** 1 Animal Systematics and Molecular Ecology Laboratory, Department of Biology, Faculty of Science, Mahidol University, Bangkok, Thailand; 2 School of Biology, Institute of Science, Suranaree University of Technology, Nakhon Ratchasima, Thailand; 3 Animal Systematics Research Unit, Department of Biology, Faculty of Science, Chulalongkorn University, Bangkok, Thailand; 4 Faculty of Fisheries, Royal University of Agriculture, Phnom Penh, Cambodia; 5 Wonders of the Mekong Project, Phnom Penh, Cambodia; 6 Research Academic and Service Office, National University of Laos, Vientiane, Laos

**Keywords:** Evolution, Phylogenetics

## Abstract

The body of knowledge regarding the classification and evolution of freshwater mussels in the family Unionidae (Bivalvia) in Indochina has recently increased. However, the taxonomic revision of all extant taxa in the region is still ongoing. In this study, the genus *Pilsbryoconcha* was revised based on an integrative analysis of shell morphology, biogeography, and molecular data. Multi-locus phylogeny indicated the availability of eight species within the genus. Four previously recognized species are *P. exilis* (Lea, 1838), *P. schomburgki* (Martens, 1860) stat. rev., *P. linguaeformis* (Morelet, 1875), and *P. carinifera* (Conrad, 1837), while four other species are described herein as *P. acuta* sp. nov., *P. mekongiana* sp. nov., *P. kittitati* sp. nov., and *P. hoikaab* sp. nov. In addition, the neotype of *P. carinifera* is also designated to clarify its long taxonomic ambiguity. Divergent time estimation and historical biogeography analysis revealed that *Pilsbryoconcha* originated in the area now called the Khorat Plateau around the middle of the Eocene (mean age = 43.12 Mya), before its range was expanded across Indochina through a series of complex geomorphological changes of river systems, which also led to diversification of the genus.

Indochina is a globally significant evolutionary hotspot, recognized for its exceptional species richness and in situ diversification of terrestrial and aquatic animals^1^. Freshwater mussels (Bivalvia: Unionoida), important benthic organisms with the ability to filter the water that surrounds them^2^, also exhibit high species diversity, with more than one hundred species being recorded in Indochinese rivers^3^, including extraordinary endemism within the tributaries of the Chao Phraya and Mekong rivers^4–12^. Despite the significant expansion of knowledge regarding the classification of Indochinese freshwater mussels in the last decade^4–12^, a comprehensive taxonomic review of all extant taxa is still incomplete. Many taxa have never received much attention, and many more hidden lineages await description^13^. In this study, we focus on the genus *Pilsbryoconcha* Simpson, 1900^14^, one of the most abundant and widely distributed freshwater mussel groups in Indochina ^15^. *Pilsbryoconcha* is characterized by its laterally thin and compressed shell, elongated linguiform shell outline, and by the lack of teeth on the hinge area^14,15^. The genus currently comprises four recognized species in the global database (i.e., MUSSELp^16^). It is known for its economic importance as a food resource^17^ and is utilized as a living filter system in fish aquaculture to reduce the amount of suspended particles^18^ and control bacteria^19^. Members of the genus are also noted for being sold as ornamental pets^20^.

This study aims to revise the systematics of the *Pilsbryoconcha* based on the integrative approach of shell morphology examination and molecular phylogenetic analysis using multi-locus markers. We also generated a time-calibrated phylogeny and prepared a series of ancestral area reconstructions, which allowed us to test the evolutionary history of *Pilsbryoconcha* in Indochinese river systems.

## Results

### Phylogeny of *Pilsbryoconcha*

**Figure 1:**
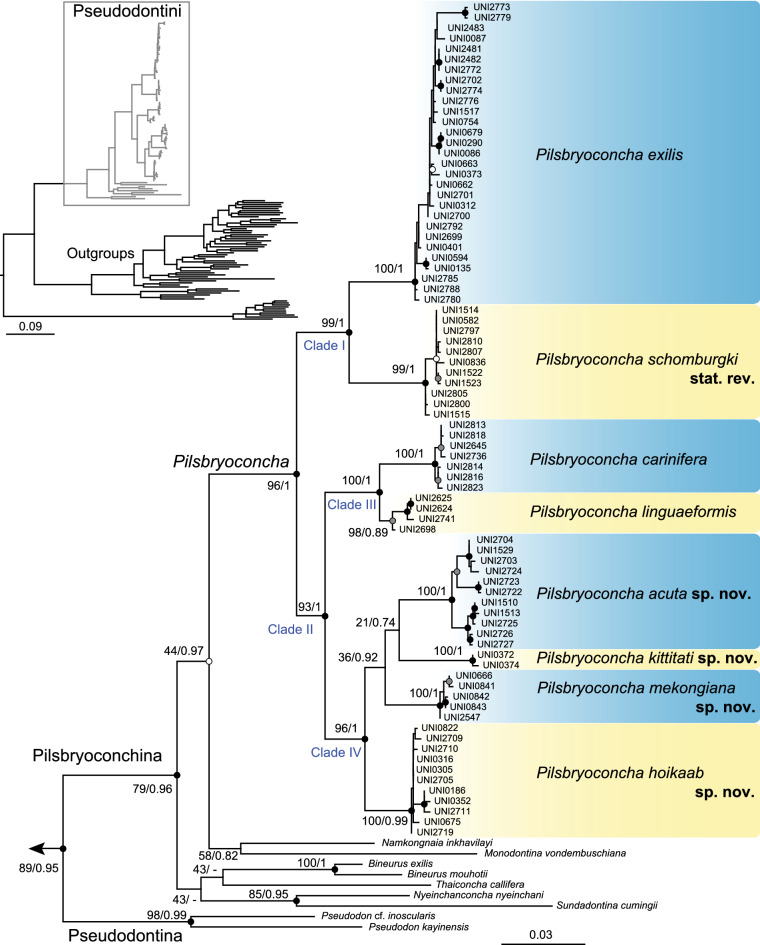
Maximum likelihood tree based on 2216 bp concatenated alignment dataset of COI + 16S + 28S genes. The outgroup sample is not shown. Numbers on nodes indicate bootstrap values (bs) from maximum likelihood (ML) and bipartition posterior probability (bpp) from Bayesian inference analysis (BI), and are shown as ML/BI. Nodes marked with black circles were sufficiently supported by both ML (bs ≥ 70) and BI (bpp ≥ 0.95). Nodes with grey circles were supported only by ML, and white circles were supported only by BI.

Multi-locus phylogenies were constructed using the Bayesian inference (BI) and maximum likelihood (ML) methods based on 2216 bp concatenated alignment dataset of mitochondrial cytochrome c oxidase subunit I (COI) and 16S large ribosomal subunit rRNA (16S rRNA), and the nuclear 28S large ribosomal subunit rDNA (28S rRNA) gene fragments. Both analyses gave similar topology and congruently retrieved *Pilsbryoconcha* as monophyletic when compared with other genera in the Pseudodontini (Fig. 1 and Supplementary Fig. S1). *Pilsbryoconcha* was recovered as two well-supported clades. The first clade (Clade I) contained two species-level subclades distributed in Java, central Thailand, and tributaries draining into the Gulf of Thailand. We assigned one subclade to *P. exilis*^21^, the type species, based on the available sequences from the topotype (Java). For another subclade, we tentatively used the available name *P. schomburgki*^22^ based on conchological similarity to the type series*.*

Another clade (Clade II) consisted of six species-level subclades. Two species, namely *P. linguaeformis*^23^ and *P. carinifera*^24^, were grouped as a sister clade with strong support (Clade III), and both were from the Tonle Sap Basin. The latter species has long been a taxonomic problem due to unavailability of type specimens and uncertain type locality^25^. Therefore, we designated the neotype herein to clarify and fix the type status. The other four lineages from the Khorat Plateau Basin were grouped as a well-supported clade (Clade IV). Each of them was separately supported as monophyly, and can be clearly distinguished from each other by conchological characters. Therefore, these four mussels from the Khorat Plateau Basin were proposed as new species and described herein.

The level of genetic divergence described by uncorrected COI p-distance among species was relatively high, ranging from 4.86 to 11.49% (8.86% average; Table 1). In contrast, intraspecific divergences were low, ranging from 0 to 1.98% (0.66% average). The average genetic divergence between the two principal clades (Clade I and II) was 9.97%.

**Table 1:** Average interspecific genetic divergence (uncorrected p-distance: % ± S.E.) between *Pilsbryoconcha* species (below diagonal) calculated from the 660 bp *COI* gene fragment sequences.

Taxa	1	2	3	4	5	6	7	8
1. *P. exilis*	**0.52 ± 0.12**							
2. *P. schomburgki*	7.94 ± 0.92	**0.20 ± 0.09**						
3. *P. linguaeformis*	8.04 ± 0.94	9.78 ± 0.88	**1.04 ± 0.28**					
4. *P. carinifera*	9.77 ± 0.89	11.49 ± 1.00	4.86 ± 0.89	**0.56 ± 0.19**				
5. *P. hoikaab* **sp. nov**	9.65 ± 1.01	10.11 ± 1.02	6.97 ± 1.09	8.82 ± 1.01	**0.58 ± 0.16**			
6. *P. mekongiana* **sp. nov**	9.83 ± 0.77	9.81 ± 0.87	7.59 ± 0.90	9.27 ± 0.97	7.04 ± 0.90	**0.45 ± 0.18**		
7. *P. kittitati* **sp. nov**	10.97 ± 1.02	11.52 ± 1.15	8.75 ± 1.08	10.35 ± 1.03	8.99 ± 1.15	7.12 ± 0.97	**0.00 ± 0.00**	
8. *P. acuta* **sp. nov**	9.24 ± 0.97	9.44 ± 0.92	8.37 ± 1.04	9.63 ± 1.01	7.50 ± 1.01	7.28 ± 1.09	8.20 ± 0.96	**1.96 ± 0.33**

Average intraspecific distances within each species are shown in bold.

### Divergent time estimation and historical biogeography

**Figure 2:**
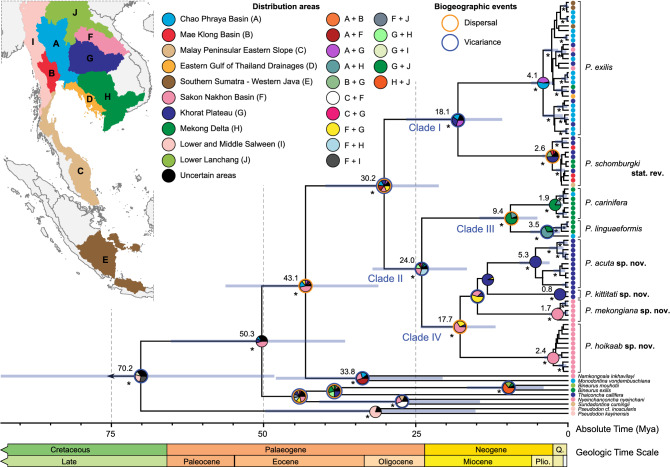
Fossil-calibrated ultrametric tree of the tribe Pseudodontini generated from BEAST v2.6.1^26^ based on the concatenated alignment dataset of COI + 16S + 28S genes. The outgroup sample is not shown. Sufficiently supported nodes (bpp ≥ 0.95) are marked with “*”. Numbers above branches are estimates of time since the most recent common ancestor (tMRCA). Node bar indicates 95% highest posterior density interval (HPD) of the node ages. Pie charts at nodes indicate probabilities of certain ancestral areas from the combined results calculated by the common area reconstruction. Border color of the pie charts indicates biogeographic events of dispersal (orange border) and vicariance (blue border) events. Map shows the boundaries of freshwater basins used in the ancestral area reconstruction analysis. Map was modified from the GIS shapefile from Freshwater Ecoregions of the World (https://www.feow.org). Geologic time scale according to the geological Society of America^81^.

Time-calibrated phylogeny reconstructed in BEAST v2.6.1^26^ and results of historical biogeography based on combined results of three analyses (S-DIVA, S-DEC, and Bayesian MCMC) generated in RASP v4.2^27,28^ are shown in Fig. 2, Supplementary Figs. S2 and S3, and summarized in Supplementary Table S2. *Pilsbryoconcha* most likely separated from the *Namkongnaia* + *Monodontina* clade somewhere in the modern-day Khorat Plateau around the middle of the Eocene (mean age = 43.12 Mya, 95%HPD = 31.14–56.27 Mya). The genus was then separated into two principal clades (Clade I and Clade II) during the Oligocene (mean age = 30.20 Mya, 95%HPD = 21.22–49.86 Mya). The area of the diversification probably occurred on the Khorat Plateau, by a combination of dispersal and vicariant events. The split of Clade I was presumably through a vicariance event disrupting the connection between the Chao Phraya and Khorat Plateau basins during the Miocene (mean age = 18.11 Mya, 95%HPD = 10.84–26.63 Mya). This divergence was more recent than that of the split among the six species in Clade II, which occurred via vicariance near the end of the Oligocene (mean age = 24.02 Mya, 95%HPD = 16.59–32.11 Mya). The hypothesized ancestral area was probably in the Middle and Lower Mekong Basin. Two species from the Tonle Sap Basin in the Lower Mekong were separated during the Miocene (Clade III; mean age = 17.71 Mya, 95%HPD = 11.90–24.21 Mya). In contrast, four species from the Khorat Plateau Basin underwent rapid radiation in the early Miocene (Clade IV; mean age = 17.71 Mya, 95%HPD = 11.90–24.21 Mya). Both radiations likely occurred through dispersal events. In situ radiation events have occurred more recently (Pliocene) in populations of all species, except for the *P. schomburgki* and *P. linguaeformis*, which have diversified by dispersal and vicariance events, respectively.

### Taxonomic account

Family Unionidae Rafinesque, 1820.

Subfamily Gonideinae Ortmann, 1916.

Tribe Pseudodontini Frierson, 1927.

Subtribe Pilsbryoconchina Bolotov et al., 2017^29^.

### *Genus Pilsbryoconcha* Simpson, 1900

*Pilsbryoconcha* Simpson, 1900: 587^14^.

*Pilsbryocandra* [sic] Coates, 1925: 83^29^.

*Type species*: *Anodonta exilis* Lea, 1838 by original designation^14^.

*Distribution*: Widespread in Indochina, Sumatra, Java, and Singapore^15,20,30,31^. The record from China^32^ needs further confirmation.

*Diagnosis*: *Pilsbryoconcha* can be recognized by its elongated linguiform shape, compressed and very thin shell, and hinge area without teeth. This genus has a shell similar to *Namkongnaia*^5^; but it can be distinguished by its wider shell, higher dorsal margin (creating a posterior wing in some species), and a slightly arched ventral margin.

*Remarks*: This genus contains nine species: five are previously described, and four are currently proposed and described herein.

### *Pilsbryoconcha exilis* (Lea, 1838)

Figures 3a–b, 4.

**Figure 3:**
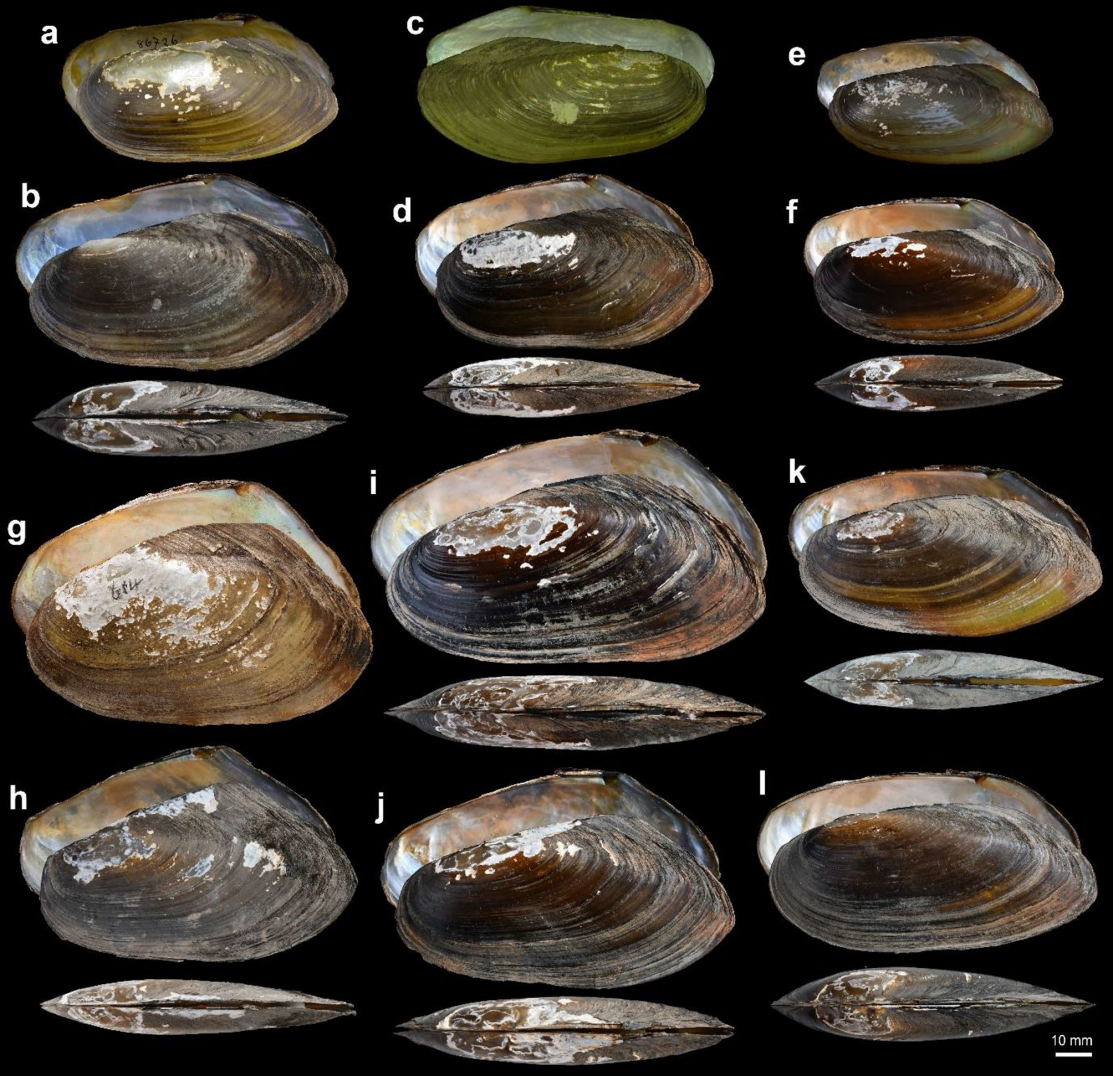
Shells of *Pilsbryoconcha* species. (**a,b**) *P. exilis*, (**a**) syntype USNM 86726, Java, (**b**) specimen MUMNH-UNI2792, Sa Kaeo, Thailand; (**c,d**) *P. schomburgki*, (**c**) holotype NHMUK 1859.5.23.8, Siam, (**d**) specimen MUMNH-UNI 2810, Nakhon Ratchasima, Thailand; (**e,f**) *P. carinifera*, (**e**) specimen ANSP 56519, ‘India’, (**f**) neotype MUMNH-UNI2823, Sa Kaeo, Thailand; (**g,h**) *P. linguaeformis*, (**g**) holotype NHMUK 1893.2.4.614, Battambang, Cambodia, (**h**) specimen MUMNH-UNI2636, Pursat, Cambodia; (**i**) *P. mekongiana* **sp. nov.** holotype MUMNH-UNI0843, Bueng Kan Thailand; (**j**) *P. hoikaab* **sp. nov.** holotype MUMNH-UNI0305, Nakhon Phanom, Thailand; (**k**) *P. acuta* **sp. nov.** holotype MUMNH-UNI1510, Ubon Ratchathani, Thailand; (**l**) *P. kittitati* **sp. nov.** holotype MUMNH-UNI0372, Udon Thani, Thailand. Scale bar = 10 mm. Image: USNM collection database [**a**], D. Graf and K Cummings, https://mussel-project.uwsp.edu/fmuotwaolcb/specimen_6006.html [**c**]; P. Callomon [**e**], and NHMUK collection database under a CC0 1.0 license [**g**].

**Figure 4:**
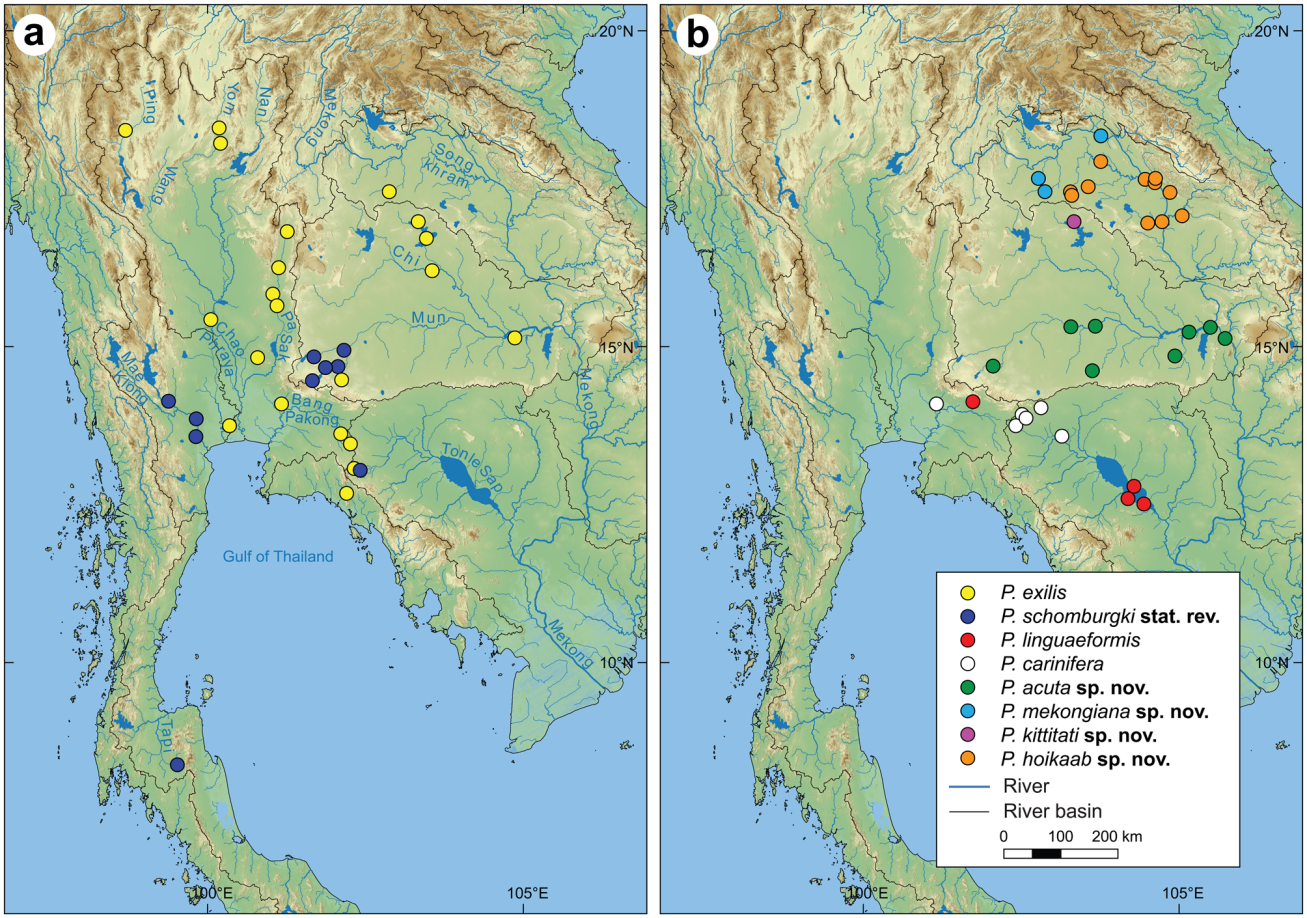
(**a,b**) Map of mainland Southeast Asia showing the distribution of *Pilsbryoconcha* species and the main river systems. Maps were developed using QGIS v3.24.3 by compiling topographic base map of freshwater river basins from the Freshwater Ecoregions of the World (https://www.feow.org), river and lake topology from the HydroSHEDS database (https://www.hydrosheds.org), and map raster data from the NASA EARTHDATA (https://www.earthdata.nasa.gov/).

*Anodonta exilis* Lea, 1836: addenda^33^. [nomen nudum].

*Anodonta exilis* Lea, 1838: 81, pl. 22, fig. 68^21^. Type locality: Java?

*Anodonta polita* Mousson, 1849: 98, pl. 19, fig. 2–3^34^. Type locality: Java.

*Spatha compressa* Martens, 1860: 16^22^. Type locality: ‘Khao-kho, north-east of Pakpriau, Siam’ [Pasak River, Khao Kho District, Phetchabun Province, Thailand].

*Monocondyloea compressa* Lea, 1863^35^: 190. Type locality: Siam [Thailand]. (not Martens, 1860^22^)

*Anodon javona* Sowerby, 1867^36^: pl. 11, sp. 33. Type locality: Java.

*Anodon kelletii* Sowerby, 1867^36^: pl. 19, sp. 71. Type locality: Unknown.

*Type*: Syntype USNM 86726, 2 shells (Fig. 3A).

Other materials examined: See Supplementary Data S1.

*Diagnosis*: This species is very similar to *P. schomburgki* but may be distinguished by a longer shell and straight ventral margin. It can also be distinguished from other congeners by several fixed nucleotide substitutions in the COI gene fragment (Table 2).

**Table 2:** Fixed nucleotide differences of COI sequences among *Pilsbryoconcha* species. Nucleotide position based on the sequence alignment in this study.

Taxa	Fixed nucleotide differences
*P. exilis*	45C, 61C, 150G, 171C, 276T, 330C, 336C, 372A, 390A, 414T, 532A, 606C, 639A
*P. schomburgki*	6A, 18A, 72A, 171T, 177G, 305A, 336A, 372C, 387C, 395C, 525G, 606T
*P. linguaeformis*	432A, 540A, 630G
*P. carinifera*	78A, 207A, 249C, 270C, 429C, 430C, 444C, 558A, 603C
*P. mekongiana* **sp. nov**	75A, 144C, 304C, 477C, 612C
*P. hoikaab* **sp. nov**	399C, 462G, 483G
*P. kittitati* **sp. nov**	102C, 180C, 192A, 285C,465C, 489C, 535C, 594C, 645G
*P. acuta* **sp. nov**	30A, 87C, 111A, 363C, 502C, 621 T

*Distribution*: In Thailand, it is distributed in the Chao Phraya, Bang Pakong, and Khorat Plateau basins, and drainages in the eastern region (Fig. 4). There are also records from Java, Laos^15^, Malay Peninsula^31^, and Singapore^20,30^.

*Remarks*: We assigned this nominal taxon to our samples based on identical shell morphology with the syntype (Fig. 3a; see Supplementary Fig. S4) and DNA sequence of topotypes (UNI2841, UNI2842, and UNI2843 in Fig. 1 from Java^5^). This is the only species known to occur in mainland Indochina and the Greater Sunda Islands (Java). Populations on the mainland have a wider shell and more prominent posterior wing (Fig. 3b). We also recognized *Spatha compressa* Martens, 1860^22^, as a junior synonym based on its current revised distribution range and genetic evidence from the topotype specimens (Pasak River, Phetchabun Province, Thailand).

### *Pilsbryoconcha schomburgki* (Martens, 1860)

Figures 3c, d, 4.

*Anodonta (Lamproscapha) schomburgki* Martens, 1860: 15^22^. Type locality: Siam [Thailand].

*Type*: Holotype NHMUK 1859.5.23.8 (Fig. 3c).

*Other materials examined*: See Supplementary Data S1.

*Diagnosis*: This species is very similar in appearance to *P. exilis*, but it may be distinguished by its shorter shell, more concave ventral margin, and broader ventral margin near the posterior portion. The two species can also be separated by several fixed nucleotide positions on the COI gene fragment (Table 2).

*Distribution*: This species is distributed only in Thailand: Mae Klong River Basin; the headwater of Mun River in the Khorat Plateau; Tapi River in the South; and Khlong Phraphut Stream, the headwater of Tonle Sap Basin (Fig. 4).

*Remarks*: The nominal name *schomburgki* was included as a valid species in *Pilsbryoconcha* by Simpson in 1900^14^. Later, it was synonymized under either *P. carinifera*^37^ or *P. exilis*^15,38^. It is resurrected here based on the conchological similarity of our specimens to the holotype (NHMUK 1859.5.23–28; Fig. 3c). This species and *P. exilis* are both distributed in the central plain of Thailand, but their ranges do not seem to overlap (Fig. 4).

### *Pilsbryoconcha carinifera* (Conrad, 1837)

Figures 3e–f, 4.

*Anodonta carinifera* Conrad, 1837: back cover^24^. Type locality: rivers in Kentucky? [probably error].

*Anodonta sempervivens* Deshayes & Jullien, 1876: 120, pl. 5, figs. 4–5^39^. Type locality: Peam Chelang, Cambodge [=Cambodia].

*Anodonta laminata* Rochebrune, 1882: 40^40^. Type locality: Rapides du Mekong.

*Type*: Neotype MUMNH-UNI2823 (length 68.6 mm, height 31.0 mm, width 14.7 mm); THAILAND: Huai Yang Stream at Nong Muang, Khok Sung District, Sa Kaeo Province; 13°52′10.3"N, 102°35′23.5"E. Topotypes: 3 shells, MUMNH-UNI2824, UNI2931 to UNI2935; same collection data as for neotype.

*Other materials examined*: See Supplementary data S1.

*Diagnosis*: This species can be distinguished from its sister taxon from the same river basin, *P. linguaeformis*, by its elongated shell outline and much lower posterior wing. It is also similar to *P. acuta* **sp. nov.** but has a less pointed posterior, more truncated posterior margin, and more prominent anterior adductor muscle. It can also be distinguished from congeners by fixed nucleotide substitutions in the COI gene fragment (Table 2).

*Description*: Shell medium-sized, thin, elongate linguiform (H/L ratio = 0.45), very inequilateral, and compressed. Dorsal margin straight to slightly curved; anterior slightly lower than posterior; posterior wing not prominent. Umbonal area eroded, not elevated and sculptured with 2 to 3 short irregular furrows. Anterior margin round; posterior margin elongated and somewhat rounded to subtruncated. Ventral margin straight to slightly curved. Posterior ridges low, wide and obtuse, and not prominent. Periostracum thin, greenish to dark brown, eroded area white to coppery-brown. Shell surface smooth with fine growth lines. Ligament long and very narrow. Hinge without dentition, or with very rudimentary pseudocardinal tooth in each valve; posterior end of hinge structure with V-shaped fossette. Anterior adductor muscle scar shallow, dropped-shape, fused with pedal retractor muscle scars; posterior adductor muscle scars very shallow. Pallial line very faint. Nacre bluish-white, salmon towards umbo.

*Distribution*: Based on our examination, this species is distributed in the headwater of Tonle Sap Basin in Thailand and Cambodia, and probably the Mekong River in Cambodia.

*Remarks*: This nominal species *Anodonta carinifera* Conrad, 1837^24^, was nominated based on a shell specimen sent to Conrad by his colleagues. The original description did not include an illustration, and only one set of shell measurements was given^24^. The unique name-bearing type was not explicitly designated, and the collection locality was said to be from ‘rivers in Kentucky’. Later, it was re-described^41^ and appeared on the species list of the North American Unionidae^42^. While proposing a new endemic Southeast Asian unionid genus, *Pilsbryoconcha* by Simpson (1900)^14^, the nominal name ‘*carinifera*’ was included among five members of the genus. Simpson further argued that ‘Southeastern Asia’ is probably the correct distribution range instead of North America^14^. Simpson’s view of the erroneous type locality was well known and widely accepted in later literature^3,37,38,43–45^.

Regarding the type specimen, Simpson provided the first illustration of the species based on the specimen found in Conrad’s collection at the ANSP and believed to be the type series^43^. However, no possible type specimen was found in the recent type catalogue in the ANSP collection^25^. Although specimen lot ANSP 56519 has this species name, it has no indication connected to Conrad’s type and has ‘India’ as the collection locality. Therefore, this specimen could not be considered Conrad’s type series (P. Callomon, per. com., Apr. 2022).

Although this nominal species is recognized as valid in some literature^3,16,37,38,43–45^, a unique name-bearing type could not be located, the type locality was probably an error, and no specimen was reported other than the ANSP 56519. These have been the causes of doubt about its identity and taxonomic status. Because of the uncertain origin, vague locality data ‘India’, and lack of DNA data, the ANSP 56519 specimen is deemed unsuitable for designation as a neotype. To address these uncertainties of *Anodonta carinifera* Conrad, 1837, the specimen that agrees well with the original description, measurements, and the specimen ANSP 56519 (Fig. 3e) is hereby designated as the neotype (MUMNH-UNI2823; Fig. 3f). Regarding this neotype designation, the type locality is in the headwater of Tonle Sap Basin in Khok Sung, Sa Kaeo Province, Thailand (13°52′10.3"N, 102°35′23.5"E) and OP589099, OP595930 and OP595859 are the associated COI, 16SrRNA, and 28S rRNA accession numbers, respectively.

### *Pilsbryoconcha linguaeformis* (Morelet, 1875)

Figures 3g, h, 4.

*Anodonta linguaeformis* Morelet, 1875: 329, pl. 14, Fig. 5^23^. Type locality: Cambodje [Cambodia].

*Type*: Holotype NHMUK 1893.2.4.614 (Fig. 3g).

*Other materials examined*: See Supplementary Data S1.

*Diagnosis*: Distinguished from others by its prominent high posterior wing and wider shell. It can also be recognized by fixed nucleotide substitutions in the COI gene fragment (Table 2).

*Distribution*: Tonle Sap Lake and its tributaries in Cambodia^15,46^, and small populations in the headwater of Bang Pakong River in eastern Thailand (Fig. 4).

*Remarks*: This nominal species was once listed as a junior synonym under *P. carinifera* by Haas (1920)^38^, or subspecies of *P. exilis* by Brandt^15^. Recently, it was resurrected as a valid species by Ng et al.^46^.

### *Pilsbryoconcha mekongiana* Jeratthitikul & Prasankok sp. nov

Figures 3I, 4. LSID: https://zoobank.org/urn:lsid:zoobank.org:act:0D40DEA8-C1F3-49B7-92E2-C0241D2D3CC4

*Type materials*: Holotype: MUMNH-UNI0843 (length 106.2 mm, height 52.5 mm, width 18.8 mm); THAILAND: Tributary of Mekong River at Khok Kong Mueang District, Bueng Kan Province; 18°20′17.4"N, 103°45′44.7"E. Paratypes: 4 shells, MUMNH-UNI0840, UNI0841, UNI0842 and UNI0844; THAILAND: same collection data as for holotype.

*Other materials examined*: See Supplementary Data S1.

*Etymology*: The specific name is from its type locality, the Mekong River.

*Diagnosis*: The new species is most similar to *P. hoikaab* **sp. nov.** but can be distinguished by having a longer shell, and a more rounded posterior end. It also bears a set of unique fixed nucleotide substitutions in COI gene fragment (Table 2).

*Description*: Shell large-sized, thin, elongately linguiform (H/L ratio = 0.47–0.50), very inequilateral, and compressed. Dorsal margin straight to slightly curved near posterior; anterior low, gradually elevated to posterior; posterior wing low. Umbonal area eroded, not elevated, and sculptured with 3–5 short irregular furrows. Anterior margin round; posterior margin truncated, somewhat curved, rounded at posterior end. Ventral margin straight, slightly concave in middle. Posterior ridges low, wide and obtuse, not prominent. Periostracum thin, greenish to dark brown, eroded part white to coppery-brown. Shell surface with fine to rough growth lines, rougher on posterior slope. Ligament long, very narrow. Hinge without dentition, or with very rudimentary pseudocardinal tooth in each valve; posterior end of hinge structure with V-shaped fossette. Anterior adductor muscle scar shallow, dropped-shape to ovate, fused with pedal retractor muscle scars; posterior adductor muscle scars very shallow. Pallial line very faint. Nacre bluish-white, salmon towards umbo.

*Distribution*: Tributaries of Mekong River in Sakon Nakhon Basin, Thailand (Fig. 4).

### *Pilsbryoconcha hoikaab* Jeratthitikul & Prasankok sp. nov

Figures 3J, 4. LSID: https://zoobank.org/urn:lsid:zoobank.org:act:15959699-CA56-41B7-94C6-8C833E747D69

*Type materials*: Holotype: MUMNH-UNI0305 (length 92.3 mm, 47.3 height mm, width 15.3 mm); THAILAND: Kam River at Na Khu, Na Kae District, Nakhon Phanom Province; 16°57′29.2"N, 104°30′16.3"E. Paratype: 1 shell, MUMNH-UNI0306; THAILAND: same collection data as for holotype.

*Other materials examined*: See Supplementary Data S1.

*Etymology*: The specific name ‘*hoikaab*’ means ‘unionids or freshwater mussels’ in the Thai language.

*Diagnosis*: This species resembles *P. mekongiana* **sp. nov.**, but it can be distinguished by its shorter shell and more truncated posterior margin with a somewhat pointed posterior end. It can also be distinguished from the congeners by fixed nucleotide substitutions in the COI gene fragment (Table 2).

*Description*: Shell medium-sized, thin, elongately linguiform (H/L ratio = 0.51), very inequilateral, and compressed. Dorsal margin straight; anterior end low, gradually elevated to posterior end; posterior wing low. Umbonal area eroded, not elevated, and sculptured with 3–5 short irregular furrows, seen only in young specimens. Anterior margin round; posterior margin truncated, pointed at posterior end. Ventral margin straight or minutely concave in middle, slightly broader posteriorly. Posterior ridges low, wide and obtuse, not prominent. Periostracum thin, greenish to dark brown, eroded part white to coppery-brown. Shell surface with fine to rough growth lines. Ligament long, very narrow. Hinge without dentition, or with very rudimentary pseudocardinal tooth in each valve; posterior end of hinge structure with V-shaped fossette. Anterior adductor muscle scar very shallow, dropped-shape, fused with pedal retractor muscle scars; posterior adductor muscle scars very shallow, almost invisible. Pallial line very faint. Nacre bluish-white, salmon towards umbo.

*Distribution*: Songkhram River and tributaries of the middle Mekong River in Thailand and Laos (Fig. 4).

### *Pilsbryoconcha acuta* Jeratthitikul & Prasankok sp. nov

Figures 3k, 4. LSID: https://zoobank.org/urn:lsid:zoobank.org:act:2631A115-9BDA-4BF9-AC9A-EB7DEB9FAEB2

*Type materials*: Holotype: MUMNH-UNI1510 (length 83.6 mm, height 39.1 mm, width 16.1 mm); THAILAND: Dom Yai River at Pho Sai, Phibun Mangsahan District, Ubon Ratchathani Province; 15°13′51.9"N, 105°09′25.4"E. Paratype: 1 shell, MUMNH-UNI1509; THAILAND: same collection data as for holotype.

*Other materials examined*: See Supplementary data S1.

*Etymology*: The specific name ‘*acuta*’ is from the Latin word meaning ‘sharp or pointed’, which refers to the pointed posterior end, the diagnostic character of this new species.

*Diagnosis*: Shell elongated, pointed posterior end and yellowish periostracum. It can also be distinguished from the others by fixed nucleotide substitutions in the COI gene fragment (Table 2).

*Description*: Shell medium-sized, thin, elongately linguiform (H/L ratio = 0.47–0.50), very inequilateral, and compressed. Dorsal margin straight to slightly curved; anterior slightly lower than posterior; posterior wing not prominent. Umbonal area eroded, not elevated, and sculptured with 3–5 short irregular furrows. Anterior margin round; posterior margin elongated and pointed. Ventral margin curved to straight. Posterior ridges low, wide and obtuse, not prominent. Periostracum thin, yellowish to green, more yellowish in young specimens, eroded part white to coppery-brown. Shell surface smooth with fine growth lines. Ligament long, very narrow. Hinge without dentition, or with very rudimentary pseudocardinal tooth in each valve; posterior end of hinge structure with V-shaped fossette. Anterior adductor muscle scar shallow, dropped-shape, fused with pedal retractor muscle scars; posterior adductor muscle scars very shallow, almost invisible. Pallial line very faint. Nacre bluish-white, creamy towards umbo.

*Distribution*: Mun River in Thailand, tributaries of Mekong River in southern Laos (Fig. 4), and probably Vietnam^7^.

*Remarks***: **Specimens from Vietnam identified as ‘*Pilsbryoconcha lemeslei*’ in Bolotov et al. (2020)^7^ are phylogenetically placed in this new species.

### *Pilsbryoconcha kittitati *Jeratthitikul & Prasankok sp. nov

Figures 3l, 4. LSID: https://zoobank.org/urn:lsid:zoobank.org:act:C25FEA1B-DE23-4946-9ED5-4B10CCB900FA

*Type materials*: Holotype: MUMNH-UNI0372 (length 90.7 mm, height 40.4 mm, width 21.3 mm); THAILAND: Unnamed pond near Nong Ya Sai, Wang Sam Mo District, Udon Thani Province; 16°58′47.4"N, 103°20′13.4"E. Paratype: 1 shell, MUMNH-UNI0374; THAILAND: same collection data as for holotype.

*Etymology*: This species name is dedicated to our colleague, Mr. Kittitat Wisittikoson, who collected these specimens.

*Diagnosis*: More elongated shell outline, more laterally inflated, and dorsal margin almost parallel with ventral margin. It is also unique in its fixed nucleotide substitutions in the COI gene fragment (Table 2).

*Description*: Shell medium-sized, thin, somewhat narrow and elongated (H/L ratio = 0.45–0.49), very inequilateral, and slightly inflated. Dorsal margin straight to slightly curved; anterior at same level as posterior; posterior wing not prominent. Umbonal area eroded, little elevated, and sculptured with 2–3 concentric furrows. Anterior margin round; posterior margin elongated and rounded. Ventral margin straight to slightly curved, almost parallel with dorsal margin. Posterior ridges moderate, wide and obtuse, not prominent. Posterior slope moderate steep near umbo. Periostracum thin, dark brown, eroded part coppery-brown. Shell surface smooth with fine growth lines. Ligament long, very narrow. Hinge without dentition, or with very rudimentary pseudocardinal tooth in each valve; posterior end of hinge structure with V-shaped fossette. Anterior adductor muscle scar shallow, ovate, fused with pedal retractor muscle scars; posterior adductor muscle scars very shallow. Pallial line very faint. Nacre bluish-white, salmon towards umbo.

*Distribution*: Known only from the type locality.

*Remarks*: This new species is especially rare. We described this species based on only two shells collected from an isolated man-made pond near a paddy field. The closest natural waterway (Wang Huea Stream) is located about 2 km away from the pond. This stream is a headwater of the Pao River, a tributary of the Chi River. A more intensive survey is necessary to assess the actual abundance and distribution range. The characteristic of the elongated shell makes this new species more conchologically similar to the *Namkongnaia*^5^. However, it can be easily distinguished by its much shorter shell (H/L ratio = 0.45 vs 0.39).

### *Pilsbryoconcha expressa* (Martens, 1900)

*Anodonta expressa* Martens, 1900: 12–13^47^. Type locality: See Danau-Baru, Indragiri, Sumatra.

*Type*: Syntype NHMUK 1901.6.14.3^48^.

*Diagnosis*: This species is conchologically similar to *P. exilis* but can be distinguished by its higher posterior half of the shell^43^.

*Distribution*: Sumatra^47^.

*Remarks*: So far, no additional specimens are available for DNA examination. Therefore, we recognized this species as valid by following the previous literature^3^. In addition, the elongate linguiform, compressed and thin shell, and lack of hinge teeth suggest that this species is more closely related to *Pilsbryoconcha* than *Namkongnaia*.

## Discussion

This study revealed *Pilsbryochoncha* from Indochina to comprise eight species-level clades. All species were recovered as monophyletic but exhibit similar shell morphology and are difficult to distinguish, especially among young specimens. The presence of morphologically indistinguishable but genetically distant species has been reported in several unionid mussel genera^4,8,12^, and might be qualified the differences in shell shape variation by geometric morphometric techniques^10,49^. However, genetic distinction based on a unique set of fixed nucleotide differences in COI sequences in each species can be used in their identification, i.e., through DNA barcoding^50^ and PCR–RFLP^51^. In our case, COI divergences among *Pilsbryoconcha* were relatively high, 4.7–11.5% (8.9% average), and comparable to other Indochinese unionids, e.g., 3.6–10.0% in *Lens*^10,52^, 6.5–12.3% in *Ensidens*^8^, 6.2–9.9% in *Hyriopsis*^6^, 5.1% in *Namkongnaia*^5^ and 2.0–10.9% in *Scabies*^9^.

The river systems in Indochina have undergone a series of complex geomorphological changes^53,54^ that certainly affected species diversification and population structure of freshwater taxa in the region^55–57^. In the case of *Pilsbryoconcha*, time-calibrated phylogeny and ancestral area reconstruction suggested its origin likely occurred in the vicinity of the present-day Khorat Plateau around 43.1 Mya in the Eocene (Fig. 2), during the same period of rapid radiation events among subtribe Pilsbryoconchina genera (approximately during the Late Cretaceous to Eocene times)^5,58^. The area around the Middle Mekong is was noted for its high species-level diversity of unionids, especially for the Rectidentini, Contradentini, and Pseudodontini^3,4,12,16^, and thus undoubtedly represented one of the ancient evolutionary hotspots for unionid radiation^59^. The transcontinental Mekong River, as in its present course, probably did not exist before 17–15 Mya, in the middle Miocene^53,54^ but instead existed as a proto-Mekong River with restricted drainages in the middle reach of the present-day Mekong River since for at least 25 Mya^53^, or even earlier, since the Early Cretaceous^60^.

The common ancestor of *Pilsbryoconcha* then was split around 30.2 Mya during the Oligocene into two deeply divergent clades (Figs. 1 and 2). Clade I was mainly distributed in central Thailand and tributaries around the Gulf of Thailand, Java, and some populations in the Khorat Plateau Basin, while Clade II was restricted to the Middle and Lower Mekong basins. This general biogeographic trend of separation between the Chao Phraya and Mekong basins has been documented in unionid mussels^4,6,8^ and other groups of freshwater taxa^56,57^. Our results suggest that the ancestral area of Clade I was likely in the area of the connection between Chao Phraya and Khorat Plateau basins. Therefore, the divergence between the two drainages could be hypothesized as a vicariance event after the ancient river split or as a colonization event from the proto-Mekong populations to the proto-Chao Phraya River. At that time, the proto-Chao Phraya River existed as a large river draining southwards from the India–Asia collision zone towards the Gulf of Thailand^61^. The colonization likely occurred via the Mun River in the Khorat Plateau, which was thought to be once connected to the proto-Chao Phraya River in the east-to-west direction before it changed flow to the present-day west-to-east direction^62,63^. The stream capture is evidenced in this study by the disjunct distribution of *P. schomburgi,* which was found in the headwaters of the Mun River and the Mae Klong River Basin. Furthermore, biogeographic inference also suggested the Khorat Plateau Basin as its origin (Fig. 2). Similar evidence can be observed in *P. exilis* (Fig. 4). However, in this case, we suspect that its unusual distribution may have been facilitated by the subsequent re-arrangement of the mainstream Mekong River, from its connection with the proto-Chao Phraya to its present course, since their intraspecific genetic distances are relatively shallow.

This study again highlights the extraordinarily high diversity and endemism of unionids in the Middle Mekong River. Basin-specific distribution ranges are found in the Khorat Plateau for *P. acuta* **sp. nov.**, the Songkram River for *P. hoikaab* **sp. nov.**, the headwater of Chi River for *P. kittitati* **sp. nov.**, and to a lesser extent of the middle Mekong for *P. mekongiana* **sp. nov.** This radiation pattern is also evidenced independently in multiple species already examined across Rectidentini, Contradentini, and Pseudodontini^4,6,8,12^, suggesting consistent biogeographic patterns of several significant barriers to dispersal within the Middle Mekong drainages. Although there is thorough connection of drainages to the mainstem of the present-day Mekong River, the proto-Mekong River was thought to be isolated to several paleo-drainages^56^. Our results suggest the existence of at least four formerly independent river sections that were later re-aligned and united with the Mekong River. The deep genetic divergence among mussel species in these areas reflects their reproductive isolation and the lack of gene flow. Previous studies proposed the Kratie-Stung Treng area in Cambodia and the Lower Lancang area in Laos as two additional paleo-drainage sections of the Middle Mekong Basin, and some unionids are endemic to these areas, i.e., *Hyriopsis kratiensis*^4^ and *Lens novoselovi*^52^.

*Pilsbryoconcha* lineages from the Tonle Sap Basin in the Lower Mekong are phylogenetically close to those from the Middle Mekong lineages (Fig. 1), similar to what was found in the genus *Bineurus*, another member of Pseudodontini^12^. However, this biogeographic pattern contrasts with unionid mussels in the tribes Rectidentini and Contradentini (e.g., *Hyriosis*^4,6^, *Ensidens*^4,8^, *Physunio*^4^, and *Lens*^4^), in which the Lower Mekong lineages are relatively close to those of the Chao Phraya Basin. These contradictory biogeographic patterns may reflect the difference in historical radiation in Indochina River systems among different unionid tribes, and thus inviting further comparative biogeographic studies of multiple unionid taxa with extensive sampling throughout the distribution range and modern genomic techniques (e.g., RADseq derived SNPs^64,65^) to be conducted in the future.

## Methods

### Specimen sampling

Specimens of *Pilsbryoconcha* were collected from several localities in Indochinese river systems (Supplementary Table S1 and data S1). These specimens cover almost all nominal names, except the rare *P. expressa* from Sumatra. Specimens were collected by hand and subjected to euthanization by two-step methods^66^. They were first placed in a container filled with fresh water. Then, 95% (v/v) ethanol was gradually added to the container, starting from approximately 5% (v/v) concentration until the foot and adductor muscles fully relaxed. The anesthetized specimens were then moved to 70% (v/v) ethanol to complete the process and for tissue fixation. Preserved specimens were separated into soft bodies and shells. For the soft body, tissues from the foot and mantle were cut and preserved in 95% (v/v) ethanol and stored at − 20 °C for molecular analysis. The remaining soft parts were preserved in 70% (v/v) ethanol and kept together with their shells as vouchers. Animal use protocol was approved by the Faculty of Science, Mahidol University Animal Care and Use Committee, SCMU-ACUC (MUSC63-026-534).

Specimens were first identified by comparing with type series, museum shell lots, or photographs from museum collections available from the online MUSSELp database^16^, and compared with the original description from literature^14,15,21–24,35,36,40,47^. The comparisons were based on shell shape, shell size, umbo position, teeth, and adductor muscle scars. Shell dimensions were measured using a digital Vernier caliper (± 0.01 mm). Measurements of type series are presented in Supplementary Table S3.

### Molecular analysis

Total genomic DNA was extracted from a portion of foot or mantle tissue using a NucleoSpin® Tissue kit (MACHEREY–NAGEL, Germany). The quality and quantity of extracted DNA solution were checked via agarose gel electrophoresis. Two mitochondrial genes, the protein-coding cytochrome c oxidase subunit I (COI) and the large ribosomal subunit rRNA gene (16S rRNA), and the 28S large ribosomal subunit rDNA nuclear gene (28S rRNA) were amplified by polymerase chain reaction (PCR) with PCR primers and laboratory protocols as described in our earlier papers^5,6^. The PCR products were cleaned and sent for sequencing on the ABI 3730XL DNA Analyzer (BIONEER, Republic of Korea).

### Phylogenetic analyses

Sequences were edited and aligned using the ClustalW algorithm in MEGA v7.0.26^67^. The final concatenated alignment used in phylogenetic tree construction contained 2216 bp (660 bp of COI, 511 of 16S, and 1045 bp of 28S). These sequences came from 80 ingroups of *Pilsbryoconcha* specimens, along with nine representatives from all genera of the tribe Pseudodontini and 60 outgroups from several tribes in the Unionidae and Margaritiferidae (Supplementary Table S1). The best-fit model of nucleotide substitution and the best partitioning scheme were identified by PartitionFinder2 v.2.3.4^68^, using a heuristic search algorithm under the Akaike Information Criterion (AICc).

Phylogenetic trees were constructed using the maximum likelihood (ML) and Bayesian inference (BI) methods and via the online CIPRES Science Gateway^69^. The ML analysis was carried out in RAxML v8.2.10^70^. The GTRGAMMA was set as the model for all gene partitions. One thousand ML bootstrap replicates were performed to assess topology supports. The BI tree was estimated in MrBayes v3.2.6^71^. The best-fit models for each partition were GTR + I + G for the first codon position of COI, 16S, and 28S; HKY + I for the second codon position of COI; and GTR + G for the third codon position of COI, as suggested by PartitionFinder^2^. Metropolis-coupled Markov chain of Monte Carlo (MC-MCMC) was run for 10 million generations. Each MCMC consisted of two runs with four chains, one of which was heated. A data partition was applied that allowed parameters to be estimated separately for each partition. Trees were sampled every 1000th generation. Stationarity was considered to have been reached when the average standard deviation of split frequencies shown in MrBayes was less than 0.01, and average estimated sample size values (ESS) were over 100 in all parameters. The first 25% of obtained trees were discarded as burn-in. The remaining trees were used to estimate the consensus tree topology, bipartition posterior probability (bpp), and branch lengths. Bipartition posterior probabilities of 0.95 were considered statistically significant for BI, and bootstrap support values larger than 70 were considered highly supported for ML^72,73^. Support values below this significance level were not regarded as significant. In addition, uncorrected pairwise genetic distances were also calculated in MEGA v7.0.26^67^ to unveil the genetic distance among taxa and clades.

### Estimation of divergence times

The time-calibrated phylogeny was reconstructed using BEAST v2.6.1^26^ based on two reliable fossil calibration points suggested by previous studies^74,75^. The first fossil was †*Shifangella margaritiferiformis*^76^ (Unionoidea: †Shifangellidae), a putative ancestor of the Margaritiferidae and Unionidae with an estimated age in the Triassic/Jurassic boundary (201 Mya)^75^. Another fossil was †*Hadrodon jurassicus*^77^, a putative ancestral lineage of the Ambleminae – (Gonideinae + Unioninae) clade with an estimated age around Late Jurassic (155 Mya)^75^. The same multi-locus dataset as for previous phylogenetic analyses was used. The analyses were run using a lognormal relaxed clock algorithm with the Yule speciation process as a tree prior^78^. The prior setting for fossil calibrations followed Froufe et al. (2020)^75^. The evolutionary model for all partitions was set as a simplified evolutionary model (HKY)^58^. Two independent MCMCs were run for 50 million generations, with tree sampling every 1000th generation. The output files were checked for convergence diagnostics and ESS of parameters using Tracer v1.7^79^. Results from two independent runs were compiled with 25% burn-in using LogCombiner v2.6.2^26^. The ESS values for all parameters were greater than 300. The maximum clade credibility (MCC) tree was obtained using TreeAnnotator v2.6.2^26^. All calculations were performed through the online CIPRES Science Gateway^69^.

### Ancestral area reconstruction

Historical biogeography by mean of ancestral areas reconstruction was inferred using three probabilistic algorithms: Statistical Dispersal-Vicariance Analysis (S-DIVA), Statistical Dispersal-Extinction-Cladogenesis (S-DEC), and Bayesian binary MCMC analysis (BBM), as implemented in RASP v4.2^27,28^. A data set of 75,002 binary trees obtained from the combined data from two BEAST analyses was used as an input tree (see above), and the MCC tree was used as the input condense tree. The input trees were pruned to only the Pseudodontini. Ten geographical areas (river basins), from which specimens were collected, were used as present area definitions, following the river basin from the Map of Freshwater Ecoregions of the World^80^ (Fig. 2), except the Khorat Plateau Basin, which was sub-divided into the Sakon Nakhon Basin (includes Songkhram River and several small tributaries that drain into the Middle Mekong River) and the rest of the Khorat Plateau Basin (Mun and Chi rivers). The purpose of this subdivision was to reflect the biogeographic pattern of separated distribution between these two basins as evidenced in several unionid taxa^4,6,8,9^. A maximum of two areas per node was used in all analyses. All other parameters were set to default settings, except the number of cycles in MCMC analysis of the BBM was set to 500000 generations. Results from the three kinds of analyses were also combined using the “Combine Results” option of the software. These combined results were then used in the subsequent biogeographic interpretation.

### Nomenclature acts

The electronic edition of this article conforms to the requirements of the amended International Code of Zoological Nomenclature. Hence, the new names contained herein are available under that Code from the electronic edition of this article. This published work and its nomenclatural acts have been registered in ZooBank, the online registration system for the ICZN. The ZooBank LSIDs (Life Science Identifiers) can be resolved and the associated information viewed through any standard web browser by appending the LSID to the prefix “http://zoobank.org/”. The LSID for this publication is: urn:lsid:zoobank.org:pub:FC94E69B-5285-4FC0-9D2F-F91107C6DAE4. The electronic edition of this work was published in a journal with an ISSN, has been archived, and is available from PubMed Central.

## Supplementary Information

Supplementary Information.

## Data Availability

Voucher specimens, including type series of new species described in this study, are deposited in Mahidol University Museum of Natural History, Department of Biology, Faculty of Science, Mahidol University, Bangkok, Thailand (MUMNH). Types series of previously described species are available in the National Museum of Natural History, Washington, USA (USNM); Natural History Museum, London, UK (NHMUK); and Academy of Natural Sciences, Philadelphia, USA (ANSP). Nucleotide sequences obtained in this study were deposited in the GenBank database, under GenBank submission numbers OP589060–OP589130 for COI, OP595891–OP595961 for 16S, and OP595820–OP595890 for 28S (Supplementary Table S1).
