# Supplementary material for: Phylogeny and biogeography of Indochinese freshwater mussels in the genus Pilsbryoconcha Simpson, 1900 (Bivalvia: Unionidae) with descriptions of four new species
Source: Sci Rep. 2022 Nov 28;12:20458. doi: 10.1038/s41598-022-24844-9 (PMC9705414; doi:10.1038/s41598-022-24844-9)
Supplement: Supplementary file 1 — Supplementary Information. [file 41598_2022_24844_MOESM1_ESM.pdf]

SUPPLEMENTARY INFORMATION

Phylogeny and biogeography of Indochinese freshwater mussels in the genus *Pilsbryoconcha* Simpson, 1900 (Bivalvia: Unionidae) with descriptions of four new species

Ekgachai Jeratthitikul, Siwanon Paphatmethin, Chirasak Sutcharit, Peng Bun Ngor, Khamla Inkhavilay, and Pongpun Prasankok*

*Corresponding author: prasankok@sut.ac.th

CONTENTS

Supplementary Figures

Figure S1 Maximum likelihood tree based on 2,188 bp concatenated alignment dataset of COI + 16S + 28S genes of the full dataset.

Figure S2 Fossil-calibrated ultrametric tree of the full dataset generated from BEAST v2.6.1 based on the concatenated alignment dataset of COI + 16S + 28S genes.

Figure S3 Historical biogeographic events of the genus *Pilsbryoconcha* and other genera in the tribe Pseudodontini.

Figure S4 Figure of type specimen in original description of *Pilsbryoconcha exilis* (Lea, 1838) (after Lea, 1838: pl. 22, fig. 68).

Supplementary Tables

Table S1 Locality with geographic coordinates and GenBank accession numbers for specimens used in phylogenetic analysis.

Table S2 The most probable ancestral areas with the inferred biogeographic events and the mean age of the most recent common ancestor with 95% highest posterior density (HPD) of the primary clades of *Pilsbryoconcha* in Indochina.

Table S3 Shell measurements for the type series of new species.

Supplementary Data

Data S1 Other material examined

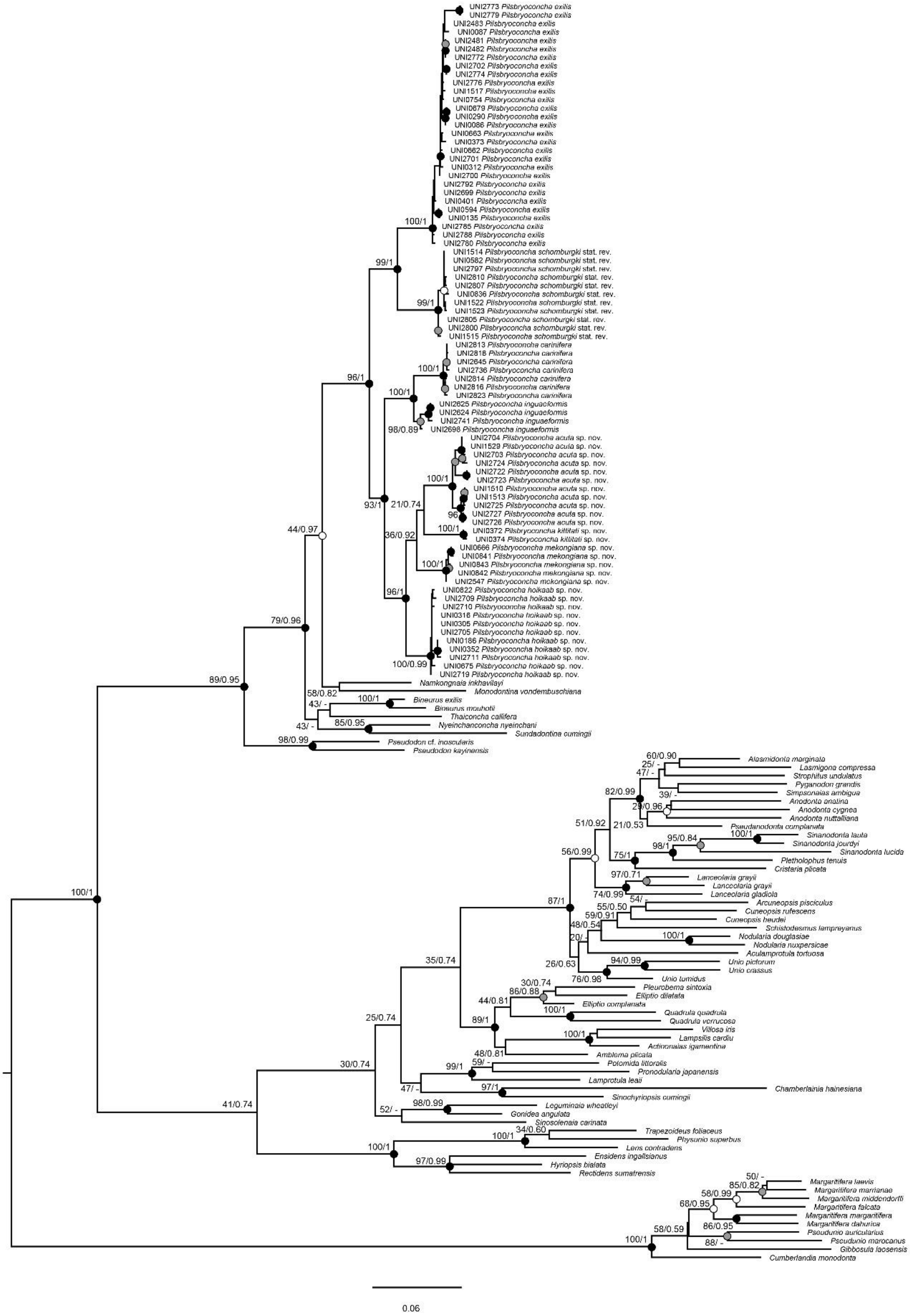

Supplementary Figure S1 Maximum likelihood tree based on 2,188 bp concatenated alignment dataset of COI + 16S + 28S genes of the full dataset. Numbers on nodes indicate bootstrap values (bs) from maximum likelihood (ML) and bipartition posterior probability (bpp) from Bayesian inference analysis (BI) and are shown as ML/BI. Nodes marked with black circles were sufficiently supported by both ML (bs ≥ 70) and BI (bpp ≥ 0.95). Nodes with grey circles were supported only by ML, and white circles were supported only by BI.

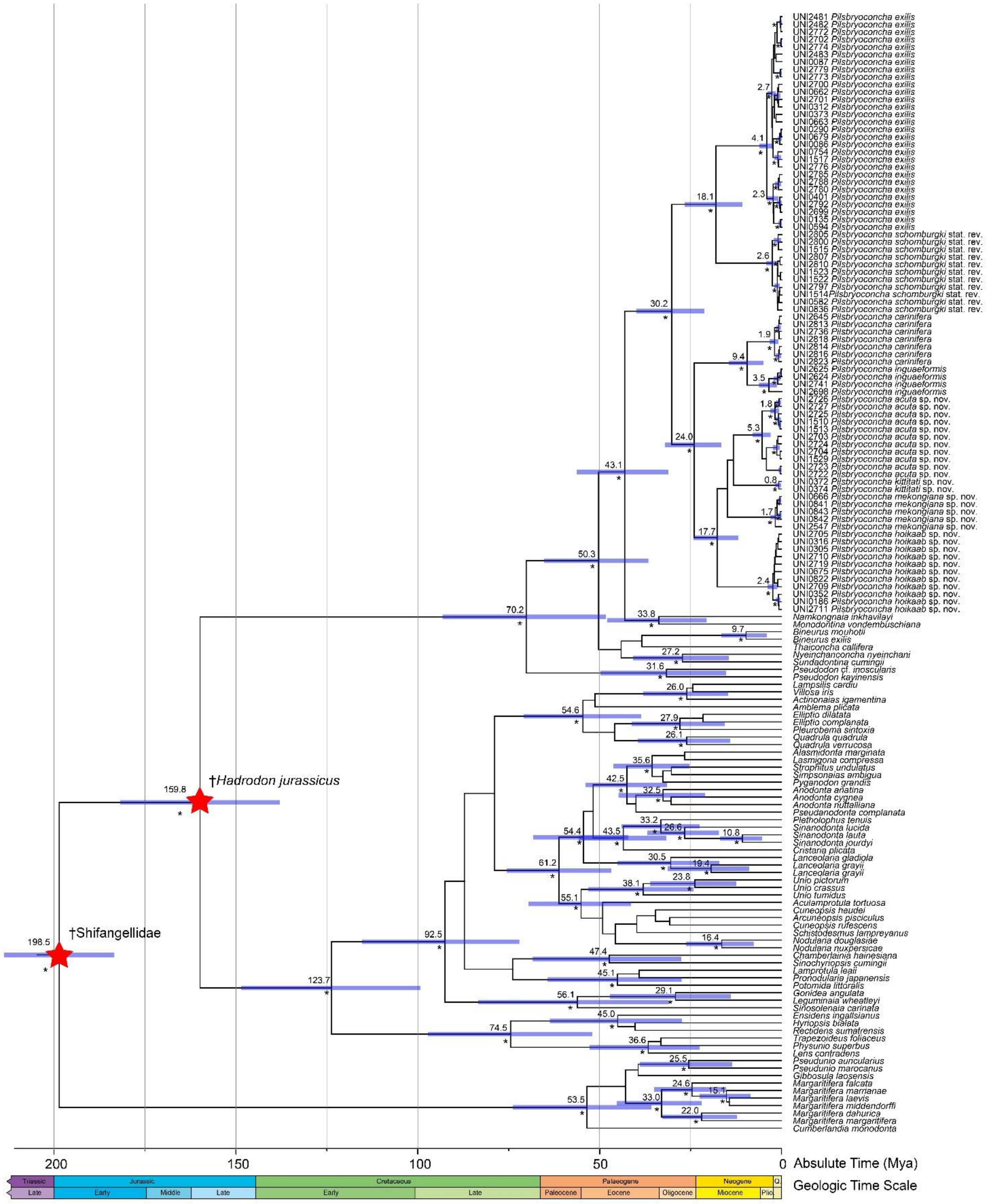

Supplementary Figure S2 Fossil-calibrated ultrametric tree of the full dataset generated from BEAST v2.6.1 based on the concatenated alignment data set of COI + 16S + 28S genes. Sufficiently supported nodes (bpp ≥ 0.95) are marked with "*". Numbers above branches are estimates of time since the most recent common ancestor (tMRCA). Node bar indicates 95% highest posterior density interval (HPD) of the node ages. Red asterisks denote fossil calibration points. Geologic time scale according to the geological Society of America.

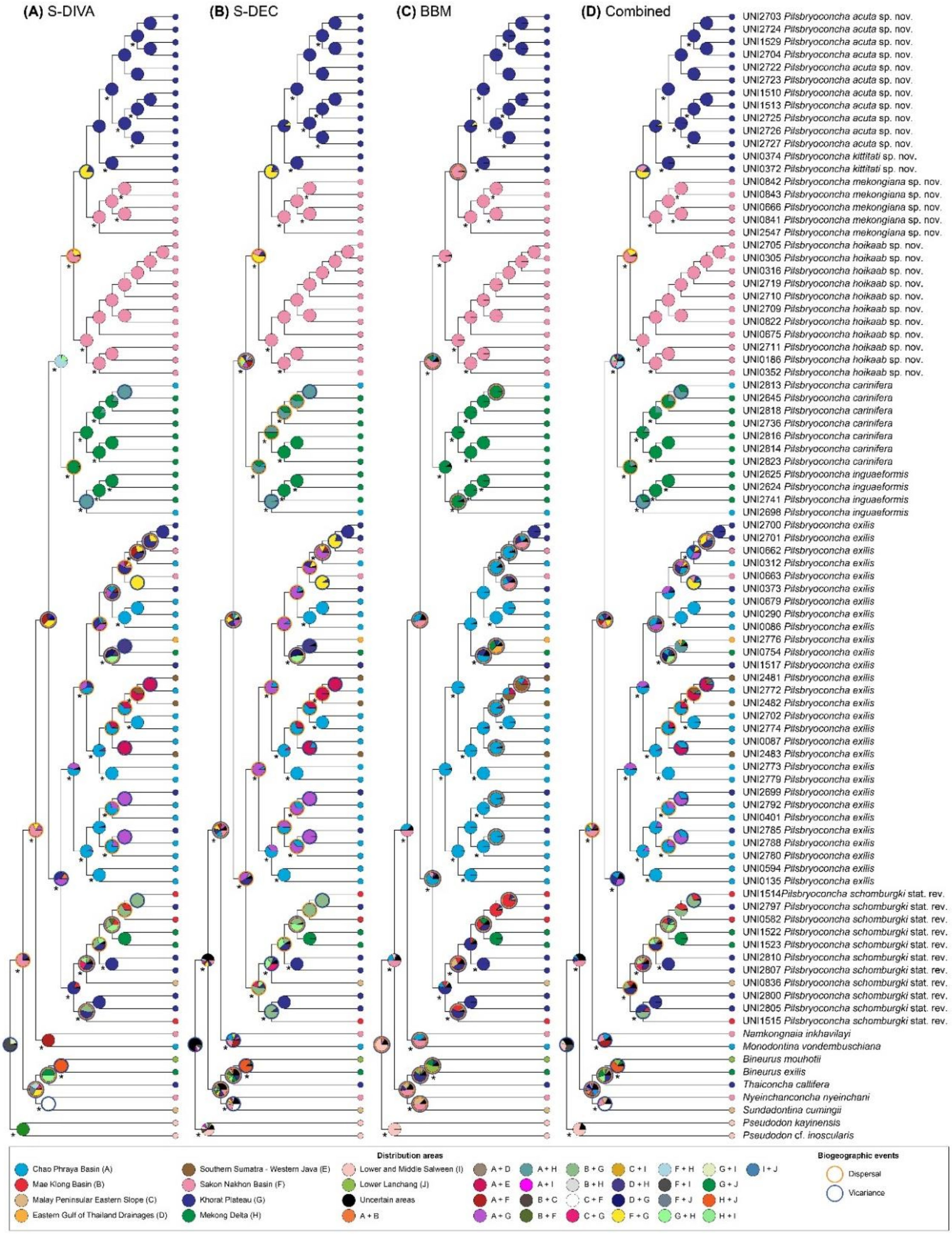

Supplementary Figure S3 Historical biogeographic events of the genus *Pilsbryoconcha* and other genera in the tribe Pseudodontini inferred from three different probabilistic algorithms: (**A**) Statistical Dispersal-vicariance (S-DIVA), (**B**) Statistical Dispersal-Extinction-Cladogenesis (S-DEC), and (**C**) Bayesian binary MCMC analysis (BBM), and (**D**) the combined results from the three analyses, as implemented in RASP v4.2. Colored circles at tips of nodes indicate the recent distribution range of each sample (see map in fig 2). Pie charts at internal nodes indicate probabilities of certain ancestral areas. Border color of the pie charts indicates biogeographic events of dispersal (orange border) and vicariance (blue border) events. Phylogenetic trees are fossil-calibration ultrametric trees generated from BEAST v2.6.1 based on the concatenated alignment data set of COI + 16S + 28S genes. Sufficiently supported nodes (bpp ≥ 0.95) are marked with "*".

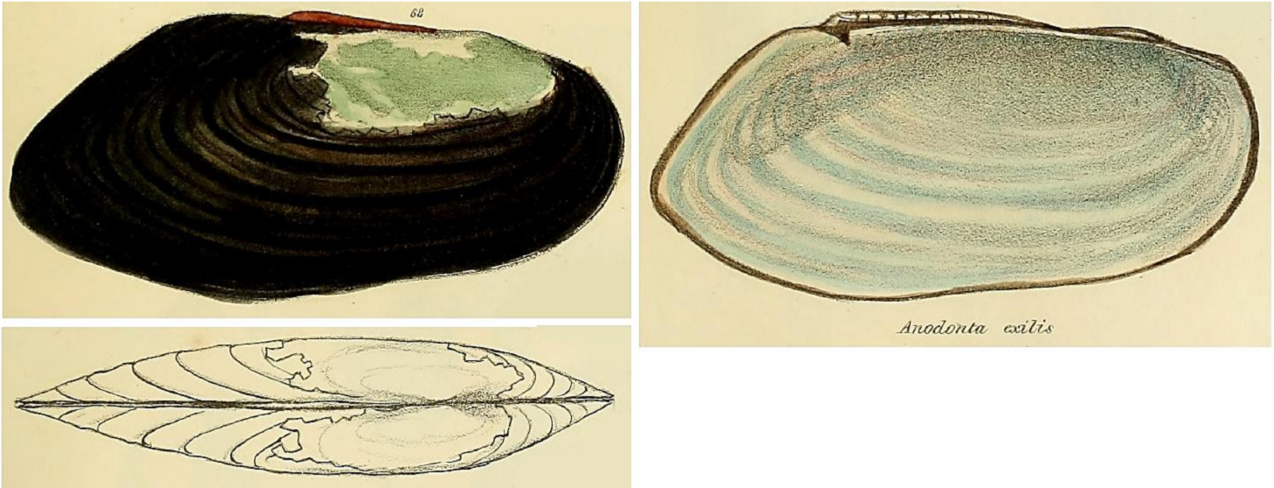

Supplementary Figure S4 Figures of type specimen in the original description of *Anodonta exilis* Lea, 1838 (after Lea, 1838: pl. 22, fig. 68).

Lea, I. (1838). "Description of new freshwater and land shells." Transactions of the American Philosophical Society 6: 1–154.

Supplementary Table S1 Locality with geographic coordinates and GenBank accession numbers for specimens used in phylogenetic analysis. The distribution areas (Area) used in ancestral area reconstruction are Chao Phraya Basin (A), Mae Klong Basin (B), Malay Peninsular Eastern Slope Drainages (C), Eastern Gulf of Thailand Drainages (D), Southern Sumatra-Western Java Drainages (E), Sakon Nakhon Basin (F), Khorat Plateau Basin (G), Mekong Delta (H), Lower and Middle Salween Basin (I), and Lower Lanchang Basin (J).

Taxa	Voucher ID	Localities	Area	Geographic coordinates	Genbank acession			Remarks
					COI	16S	28S	
**Family UNIONIDAE Rafinesque, 1820**								
**Subfamily GONIDEINAE Ortmann, 1916**								
**Tribe PSEUDODONTINI Frierson, 1927**								
**Subtribe PILSBRYOCONCHINA Bolotov et al., 2017**								
**Ingroups**								
Pilsbryoconcha exilis (Lea, 1838)	MUMNH-UNI2481	Indonesia: West Java Basin, West Java, Bogor Botanical Garden	E	6°35'58.4"S 106°48'09.7"E	MZ822408	MZ822908	MZ822930	
Pilsbryoconcha exilis (Lea, 1838)	MUMNH-UNI2482	Indonesia: West Java Basin, West Java, Bogor Botanical Garden	E	6°35'58.4"S 106°48'09.7"E	MZ822409	MZ822909	MZ822931	
Pilsbryoconcha exilis (Lea, 1838)	MUMNH-UNI2483	Indonesia: West Java Basin, West Java, Bogor Botanical Garden	E	6°35'58.4"S 106°48'09.7"E	MZ822410	MZ822910	MZ822932	
Pilsbryoconcha exilis (Lea, 1838)	MUMNH-UNI0312	Thailand: Chao Phraya River Basin, Lamphun, Ping River	A	18°25'30.2"N 98°42'4.4"E	OP589060	OP595891	OP595820	This study
Pilsbryoconcha exilis (Lea, 1838)	MUMNH-UNI2773	Thailand: Chao Phraya River Basin, Phrae, Song River	A	18°27'47.2"N 100°11'01.8"E	OP589061	OP595892	OP595821	This study
Pilsbryoconcha exilis (Lea, 1838)	MUMNH-UNI0086	Thailand: Chao Phraya River Basin, Phrae, Mae Lai River	A	18°13'11.0"N 100°12'24.0"E	OP589062	OP595893	OP595822	This study
Pilsbryoconcha exilis (Lea, 1838)	MUMNH-UNI0087	Thailand: Chao Phraya River Basin, Phrae, Mae Lai River	A	18°13'11.0"N 100°12'24.0"E	OP589063	OP595894	OP595823	This study
Pilsbryoconcha exilis (Lea, 1838)	MUMNH-UNI2779	Thailand: Chao Phraya River Basin, Uthai Thani, Sakae Krang River	A	15°25'43.6"N 100°03'10.9"E	MZ822412	MZ822912	MZ822934	
Pilsbryoconcha exilis (Lea, 1838)	MUMNH-UNI0401	Thailand: Chao Phraya River Basin, Wichian Buri, Pasak River	A	15°38'56.4"N 101°06'01.3"E	OP589064	OP595895	OP595824	This study
Pilsbryoconcha exilis (Lea, 1838)	MUMNH-UNI0135	Thailand: Chao Phraya River Basin, Phetchabun, Lom Sak, Unnamed Pond	A	16°49'18.3"N 101°15'43.7"E	OP589065	OP595896	OP595825	This study
Pilsbryoconcha exilis (Lea, 1838)	MUMNH-UNI0594	Thailand: Chao Phraya River Basin, Phetchabun, Bueng Sam Phan	A	15°49'58.7"N 101°02'05.3"E	OP589066	OP595897	OP595826	This study
Pilsbryoconcha exilis (Lea, 1838)	MUMNH-UNI0679	Thailand: Chao Phraya River Basin, Wichian Buri, Pasak River	A	15°38'56.4"N 101°06'01.3"E	OP589067	OP595898	OP595827	This study
Pilsbryoconcha exilis (Lea, 1838)	MUMNH-UNI0290	Thailand: Chao Phraya River Basin, Phetchabun, Pasak River	A	16°15'02.2"N 101°07'36.4"E	MZ822411	MZ822911	MZ822933	
Pilsbryoconcha exilis (Lea, 1838)	MUMNH-UNI2774	Thailand: Chao Phraya River Basin, Lop Buri, Sablek Reservoir	A	14°49'37.5"N 100°47'35.5"E	OP589068	OP595899	OP595828	This study
Pilsbryoconcha exilis (Lea, 1838)	MUMNH-UNI2702	Thailand: Chao Phraya River Basin, Lop Buri, Sablek Reservoir	A	14°49'37.5"N 100°47'35.5"E	OP589069	OP595900	OP595829	This study
Pilsbryoconcha exilis (Lea, 1838)	MUMNH-UNI2776	Thailand: Eastern Gulf of Thailand Drainages, Chanthaburi, Nong Tapong Pond	D	12°40'43.0"N 102°12'10.3"E	OP589070	OP595901	OP595830	This study
Pilsbryoconcha exilis (Lea, 1838)	MUMNH-UNI0754	Thailand: Tonle Sap Basin, Chanthaburi, Soi Dao, Sai Khao Stream	H	13°04'20.8"N 102°19'19.4"E	OP589071	OP595902	OP595831	This study

Pilsbryoconcha exilis (Lea, 1838)	MUMNH-UNI2780	Thailand: Chao Phraya River Basin, Nakhon Pathom, Mahidol University Salaya Campus	A	13°04'20.8"N 102°19'19.4"E	OP589072	OP595903	OP595832	This study
Pilsbryoconcha exilis (Lea, 1838)	MUMNH-UNI2788	Thailand: Bang Pakong River Basin, Nakhon Nayok, Mueang Canal	A	14°05'39.7"N 101°10'17.8"E	OP589073	OP595904	OP595833	This study
Pilsbryoconcha exilis (Lea, 1838)	MUMNH-UNI2792	Thailand: Bang Pakong River Basin, Sa Kaeo, Khlong Hat	A	13°27'39.5"N 102°15'46.7"E	OP589074	OP595905	OP595834	This study
Pilsbryoconcha exilis (Lea, 1838)	MUMNH-UNI2772	Thailand: Bang Pakong River Basin, Sa Kaeo, Klong Hinpun	A	13°37'18.0"N 102°06'35.1"E	OP589075	OP595906	OP595835	This study
Pilsbryoconcha exilis (Lea, 1838)	MUMNH-UNI0663	Thailand: Mekong River Basin, Udon Thani, Sam Phrao, Unnamed pond	F	17°27'15.9"N 102°52'37.8"E	OP589076	OP595907	OP595836	This study
Pilsbryoconcha exilis (Lea, 1838)	MUMNH-UNI0662	Thailand: Mekong River Basin, Udon Thani, Sam Phrao, Unnamed pond	F	17°27'15.9"N 102°52'37.8"E	OP589077	OP595908	OP595837	This study
Pilsbryoconcha exilis (Lea, 1838)	MUMNH-UNI0373	Thailand: Korat Plateau Basin, Udon Thani, Wang Sam Mo, Unnamed pond	G	16°58'47.4"N 103°20'13.4"E	OP589078	OP595909	OP595838	This study
Pilsbryoconcha exilis (Lea, 1838)	MUMNH-UNI2699	Thailand: Korat Plateau Basin, Kalasin, Lam Pao Dam, Thepsuda Bridge	G	16°42'54.7"N 103°27'58.0"E	OP589079	OP595910	OP595839	This study
Pilsbryoconcha exilis (Lea, 1838)	MUMNH-UNI1517	Thailand: Korat Plateau Basin, Kalasin, Lam Pao Dam, Thepsuda Bridge	G	16°42'54.7"N 103°27'58.0"E	OP589080	OP595911	OP595840	This study
Pilsbryoconcha exilis (Lea, 1838)	MUMNH-UNI2701	Thailand: Korat Plateau Basin, Roi Et, Chi River	G	16°12'10.2"N 103°33'23.7"E	OP589081	OP595912	OP595841	This study
Pilsbryoconcha exilis (Lea, 1838)	MUMNH-UNI2785	Thailand: Korat Plateau Basin, Nakhon Ratchasima Lam Mun bon Dam,	G	14°28'27.9"N 102°07'25.9"E	OP589082	OP595913	OP595842	This study
Pilsbryoconcha exilis (Lea, 1838)	MUMNH-UNI2700	Thailand: Korat Plateau Basin, Ubon Ratchathani, Warin Chamrap, Unnamed pond	G	15°08'20.5"N 104°51'59.4"E	OP589083	OP595914	OP595843	This study
Pilsbryoconcha schomburgki (Martens, 1860) **stat. rev.**	MUMNH-UNI1515	Thailand: Mae Klong Basin, Kanchanaburi, Taphoen Stream	B	14°08'13.8"N 99°22'59.6"E	OP589084	OP595915	OP595844	This study
Pilsbryoconcha schomburgki (Martens, 1860) **stat. rev.**	MUMNH-UNI1514	Thailand: Mae Klong Basin, Ratchaburi, Ban Pong, Mae Klong River	B	13°51'28.7"N 99°49'19.1"E	OP589085	OP595916	OP595845	This study
Pilsbryoconcha schomburgki (Martens, 1860) **stat. rev.**	MUMNH-UNI0582	Thailand: Mae Klong Basin, Ratchaburi, Mueang, Mae Klong River	B	13°34'34.4"N 99°48'59.7"E	OP589086	OP595917	OP595846	This study
Pilsbryoconcha schomburgki (Martens, 1860) **stat. rev.**	MUMNH-UNI2797	Thailand: Korat Plateau Basin, Nakhon Ratchasima, Nong Ta Khong Pond	G	14°56'38.3"N 102°09'38.0"E	OP589087	OP595918	OP595847	This study
Pilsbryoconcha schomburgki (Martens, 1860) **stat. rev.**	MUMNH-UNI2800	Thailand: Korat Plateau Basin, Nakhon Ratchasima, Pak Thong Chai, Unnamed Stream	G	14°41'07.8"N 102°03'55.4"E	OP589088	OP595919	OP595848	This study
Pilsbryoconcha schomburgki (Martens, 1860) **stat. rev.**	MUMNH-UNI2805	Thailand: Korat Plateau Basin, Nakhon Ratchasima, Sabpradu Reservoir	G	14°50'20.1"N 101°41'06.8"E	OP589089	OP595920	OP595849	This study
Pilsbryoconcha schomburgki (Martens, 1860) **stat. rev.**	MUMNH-UNI1522	Thailand: Tonle Sap Basin, Chanthaburi, Pong Nam Ron, Phraphut Stream	H	13°02'39.2"N 102°25'05.1"E	OP589090	OP595921	OP595850	This study
Pilsbryoconcha schomburgki (Martens, 1860) **stat. rev.**	MUMNH-UNI1523	Thailand: Tonle Sap Basin, Chanthaburi, Pong Nam Ron, Phraphut Stream	H	13°02'39.2"N 102°25'05.1"E	OP589091	OP595922	OP595851	This study
Pilsbryoconcha schomburgki (Martens, 1860) **stat. rev.**	MUMNH-UNI2807	Thailand: Korat Plateau Basin, Nakhon Ratchasima, Lam Samlai Reservoir	G	14°40'09.1"N 101°52'05.4"E	OP589092	OP595923	OP595852	This study
Pilsbryoconcha schomburgki (Martens, 1860) **stat. rev.**	MUMNH-UNI2810	Thailand: Korat Plateau Basin, Nakhon Ratchasima, Lam Phra Phloeng Stream	G	14°27'43.5"N 101°39'38.0"E	OP589093	OP595924	OP595853	This study
Pilsbryoconcha schomburgki (Martens, 1860) **stat. rev.**	MUMNH-UNI0836	Thailand: Malay Peninsular Eastern Slope Drainages, Nakhon Si Thammarat, Min Stream	C	8°22'52.5"N 99°31'22.3"E	OP589094	OP595925	OP595854	This study
Pilsbryoconcha linguaeformis (Morelet, 1875)	MUMNH-UNI2625	Cambodia: Tonle Sap Basin, Tonle Sap Lake, Boeng Tonle Chhma	H	12°47'25.1"N 104°17'55.7"E	MZ822413	MZ822913	MZ822935	
Pilsbryoconcha linguaeformis (Morelet, 1875)	MUMNH-UNI2624	Cambodia: Tonle Sap Basin, Tonle Sap Lake, Chhnok Tru	H	12°30'36.5"N 104°27'18.2"E	MZ822414	MZ822914	MZ822936	

Pilsbryoconcha linguaeformis (Morelet, 1875)	MUMNH-UNI2741	Cambodia: Tonle Sap Basin, Tonle Sap Lake open area	H	12°35'42.1"N 104°12'13.1"E	MZ822415	MZ822915	MZ822937	
Pilsbryoconcha linguaeformis (Morelet, 1875)	MUMNH-UNI2698	Thailand: Bang Pakong River Basin, Prachin Buri, Hanuman River	A	14°07'44.8"N 101°44'50.3"E	MZ822416	MZ822916	MZ822938	
Pilsbryoconcha carinifera (Conrad, 1837)	MUMNH-UNI2813	Thailand: Bang Pakong River Basin, Nakhon Nayok, Mueang Canal	A	14°05'39.7"N 101°10'17.8"E	OP589095	OP595926	OP595855	This study
Pilsbryoconcha carinifera (Conrad, 1837)	MUMNH-UNI2818	Thailand: Tonle Sap Basin, Sa Kaeo, Watthana Nakhon, Yang Stream	H	13°55'31.5"N 102°31'37.7"E	OP589096	OP595927	OP595856	This study
Pilsbryoconcha carinifera (Conrad, 1837)	MUMNH-UNI2816	Thailand: Tonle Sap Basin, Sa Kaeo, Watthana Nakhon, Phrom Hot Stream	H	13°44'56.6"N 102°25'37.6"E	OP589097	OP595928	OP595857	This study
Pilsbryoconcha carinifera (Conrad, 1837)	MUMNH-UNI2814	Thailand: Tonle Sap Basin, Sa Kaeo, Ta Phraya, Yang Stream	H	14°02'00.4"N 102°49'35.4"E	OP589098	OP595929	OP595858	This study
Pilsbryoconcha carinifera (Conrad, 1837)	MUMNH-UNI2823	Thailand: Tonle Sap Basin, Sa Kaeo, Khok Sung, Yang Stream	H	13°52'10.3"N 102°35'23.5"E	OP589099	OP595930	OP595859	This study
Pilsbryoconcha carinifera (Conrad, 1837)	MUMNH-UNI2645	Cambodia: Tonle Sap Basin, Tonle Sap Lake, Preah Netr Preah	H	13°35'01.8"N 103°09'24.8"E	OP589100	OP595931	OP595860	This study
Pilsbryoconcha carinifera (Conrad, 1837)	MUMNH-UNI2736	Cambodia: Tonle Sap Basin, Tonle Sap Lake, Preah Netr Preah	H	13°35'01.8"N 103°09'24.8"E	OP589101	OP595932	OP595861	This study
Pilsbryoconcha hoikaab Jeratthitikul & Prasankok **sp. nov.**	MUMNH-UNI0822	Thailand: Sakon Nakhon Basin, Udon Thani, Thung Fon, Songkhram River	F	17°27'06.5"N 103°16'50.4"E	OP589102	OP595933	OP595862	This study
Pilsbryoconcha hoikaab Jeratthitikul & Prasankok **sp. nov.**	MUMNH-UNI0316	Thailand: Sakon Nakhon Basin, Udon Thani, Nong Han, Songkhram River	F	17°23'44.5"N 103°18'01.7"E	OP589103	OP595934	OP595863	This study
Pilsbryoconcha hoikaab Jeratthitikul & Prasankok **sp. nov.**	MUMNH-UNI0675	Thailand: Sakon Nakhon Basin, Bueng Kan, Seka, Songkhram River	F	17°55'54.7"N 103°45'36.9"E	OP589104	OP595935	OP595864	This study
Pilsbryoconcha hoikaab Jeratthitikul & Prasankok **sp. nov.**	MUMNH-UNI0186	Thailand: Sakon Nakhon Basin, Nakhon Phanom, Tha Uthen, Songkhram River	F	17°38'59.8"N 104°27'45.6"E	OP589105	OP595936	OP595865	This study
Pilsbryoconcha hoikaab Jeratthitikul & Prasankok **sp. nov.**	MUMNH-UNI2705	Thailand: Mekong River Basin, Nakhon Phanom, That Phanom, Mekong River	F	16°58'46.6"N 104°43'50.4"E	OP589106	OP595937	OP595866	This study
Pilsbryoconcha hoikaab Jeratthitikul & Prasankok **sp. nov.**	MUMNH-UNI0305	Thailand: Mekong River Basin, Nakhon Phanom, Na Kae, Kam River	F	16°57'29.2"N 104°30'16.3"E	OP589107	OP595938	OP595867	This study
Pilsbryoconcha hoikaab Jeratthitikul & Prasankok **sp. nov.**	MUMNH-UNI0352	Thailand: Mekong River Basin, Sakon Nakhon, Charoen Sin, Yam River	F	17°31'56.9"N 103°33'39.5"E	OP589108	OP595939	OP595868	This study
Pilsbryoconcha hoikaab Jeratthitikul & Prasankok **sp. nov.**	MUMNH-UNI2710	Laos: Mekong River Basin, Khammouane, Nam Hin Boun River	F	17°35'46.8"N 104°37'04.9"E	OP589109	OP595940	OP595869	This study
Pilsbryoconcha hoikaab Jeratthitikul & Prasankok **sp. nov.**	MUMNH-UNI2719	Laos: Mekong River Basin, Khammouane, Nam Pa Kan River	F	17°39'52.2"N 104°37'37.0"E	OP589110	OP595941	OP595870	This study
Pilsbryoconcha hoikaab Jeratthitikul & Prasankok **sp. nov.**	MUMNH-UNI2709	Laos: Mekong River Basin, Khammouane, Thakhek, Nam Don River	F	17°26'32.9"N 104°51'22.2"E	OP589111	OP595942	OP595871	This study
Pilsbryoconcha hoikaab Jeratthitikul & Prasankok **sp. nov.**	MUMNH-UNI2711	Laos: Mekong River Basin, Savannakhet, Xe Nou River	F	17°4'22.34"N 105°2'54.97"E	OP589112	OP595943	OP595872	This study
Pilsbryoconcha mekongiana Jeratthitikul & Prasankok **sp. nov.**	MUMNH-UNI0842	Thailand: Mekong River Basin, Bueng Kan, Khok Kong Mueang, Tributary of Mekong River	F	18°20'17.4"N 103°45'44.7"E	OP589113	OP595944	OP595873	This study
Pilsbryoconcha mekongiana Jeratthitikul & Prasankok **sp. nov.**	MUMNH-UNI0843	Thailand: Mekong River Basin, Bueng Kan, Khok Kong Mueang, Tributary of Mekong River	F	18°20'17.4"N 103°45'44.7"E	OP589114	OP595945	OP595874	This study
Pilsbryoconcha mekongiana Jeratthitikul & Prasankok **sp. nov.**	MUMNH-UNI0841	Thailand: Mekong River Basin, Bueng Kan, Khok Kong Mueang, Tributary of Mekong River	F	18°20'17.4"N 103°45'44.7"E	OP589115	OP595946	OP595875	This study
Pilsbryoconcha mekongiana Jeratthitikul & Prasankok **sp. nov.**	MUMNH-UNI0666	Thailand: Mekong River Basin, Udon Thani, Sam Phrao, Unnamed pond	F	17°27'15.9"N 102°52'37.8"E	OP589116	OP595947	OP595876	This study

Pilsbryoconcha mekongiana Jeratthitikul & Prasankok **sp. nov.**	MUMNH-UNI2547	Thailand: Mekong River Basin, Udon Thani, Sakhrai Huai Nam Suai Stream	F	17°39'50.7"N 102°46'22.3"E	OP589117	OP595948	OP595877	This study
Pilsbryoconcha kittitati Jeratthitikul & Prasankok **sp. nov.**	MUMNH-UNI0374	Thailand: Korat Plateau Basin, Udon Thani, Wang Sam Mo, Unnamed pond	G	16°58'47.4"N 103°20'13.4"E	OP589118	OP595949	OP595878	This study
Pilsbryoconcha kittitati Jeratthitikul & Prasankok **sp. nov.**	MUMNH-UNI0372	Thailand: Korat Plateau Basin, Udon Thani, Wang Sam Mo, Unnamed pond	G	16°58'47.4"N 103°20'13.4"E	OP589119	OP595950	OP595879	This study
Pilsbryoconcha acuta Jeratthitikul & Prasankok **sp. nov.**	MUMNH-UNI1529	Thailand: Korat Plateau Basin, Nakhon Ratchasima, Pak Thong Chai, Lam Phra Phloeng Stream	G	14°41'34.9"N 102°03'18.0"E	OP589120	OP595951	OP595880	This study
Pilsbryoconcha acuta Jeratthitikul & Prasankok **sp. nov.**	MUMNH-UNI2703	Thailand: Korat Plateau Basin, Surin, Chumphon Buri, Mun River	G	15°18'55.1"N 103°17'12.2"E	OP589121	OP595952	OP595881	This study
Pilsbryoconcha acuta Jeratthitikul & Prasankok **sp. nov.**	MUMNH-UNI2704	Thailand: Korat Plateau Basin, Surin, Unnamed Stream on Chok Chai - Det Udom Road	G	14°37'11.0"N 103°37'37.4"E	OP589122	OP595953	OP595882	This study
Pilsbryoconcha acuta Jeratthitikul & Prasankok **sp. nov.**	MUMNH-UNI2724	Thailand: Korat Plateau Basin, Surin, Tha Tum, Mun River	G	15°19'28.7"N 103°40'28.2"E	OP589123	OP595954	OP595883	This study
Pilsbryoconcha acuta Jeratthitikul & Prasankok **sp. nov.**	MUMNH-UNI1510	Thailand: Korat Plateau Basin, Ubon Ratchathani, Phibun Mangsahan, Dom Yai River	G	15°13'51.9"N 105°09'25.4"E	OP589124	OP595955	OP595884	This study
Pilsbryoconcha acuta Jeratthitikul & Prasankok **sp. nov.**	MUMNH-UNI1513	Thailand: Korat Plateau Basin, Ubon Ratchathani, Khong Chiam, Tributary of Mun River	G	15°18'26.1"N 105°29'44.9"E	OP589125	OP595956	OP595885	This study
Pilsbryoconcha acuta Jeratthitikul & Prasankok **sp. nov.**	MUMNH-UNI2725	Thailand: Korat Plateau Basin, Ubon Ratchathani, Det Udom, Unnamed stream	G	14°51'11.8"N 104°56'02.8"E	OP589126	OP595957	OP595886	This study
Pilsbryoconcha acuta Jeratthitikul & Prasankok **sp. nov.**	MUMNH-UNI2727	Thailand: Korat Plateau Basin, Ubon Ratchathani, Det Udom, Unnamed stream	G	14°51'11.8"N 104°56'02.8"E	OP589127	OP595958	OP595887	This study
Pilsbryoconcha acuta Jeratthitikul & Prasankok **sp. nov.**	MUMNH-UNI2726	Thailand: Korat Plateau Basin, Ubon Ratchathani, Det Udom, Unnamed stream	G	14°51'11.8"N 104°56'02.8"E	OP589128	OP595959	OP595888	This study
Pilsbryoconcha acuta Jeratthitikul & Prasankok **sp. nov.**	MUMNH-UNI2722	Laos: Mekong River Basin, Champasak, Pakse, Unnamed pond	G	15°07'48.7"N 105°43'44.3"E	OP589129	OP595960	OP595889	This study
Pilsbryoconcha acuta Jeratthitikul & Prasankok **sp. nov.**	MUMNH-UNI2723	Laos: Mekong River Basin, Champasak, Pakse, Unnamed pond	G	15°07'48.7"N 105°43'44.3"E	OP589130	OP595961	OP595890	This study
**Outgroups**								
Monodontina vondembuschiana (Lea, 1840)	BIV1822	Malaysia	A	n/a	MK994774	MK994775	n/a	
Bineurus mouhotii (Lea, 1863)	RMBH biv0182_2	Laos: Mekong River basin, Nam Long River	J	n/a	KX865876	KX865647	KX865747	
Bineurus exilis (Morelet, 1866)	MUMNH-UNI2728	Cambodia: Tole Sap basin, Sangker River, Battambang	H	n/a	MZ822405	MZ822905	MZ822927	
Thaiconcha callifera (Martens, 1860)	RMBH biv0120_3	Thailand: Mekong River basin, Phong River	G	n/a	KX865865	KX865636	KX865737	
Sundadontina cumingii (Lea, 1850)	X115	Malaysia	C	n/a	KX051295	n/a	n/a	
Nyeinchanconcha nyeinchani Bolotov et al., 2020	UMMZ 304648	Laos: Mekong River basin, Nam Phiat River	F	n/a	KP795025	KP795050	KP795008	
Namkongnaia inkhavilayi Jeratthitikul et al., 2021	MUMNH-UNI2704	Laos: Mekong River basin, Local market near Xe Bangfai River	F	n/a	MZ822395	MZ822895	MZ822917	
**Subtribe PSEUDODONTINA Frierson, 1927**								
Pseudodon cf. *inoscularis* (Gould, 1844)	RMBH biv0110_5	Myanmar: Ayeyarwady River basin, a tributary of Lake Indawgyi	I	n/a	KX865858	KX865629	KX865730	
Pseudodon kayinensis Bolotov et al., 2020	RMBH biv0618_3	Myanmar: Ataran River basin, Winyaw River	I	n/a	MN275045	MN307240	MN307181	
**Tribe GONIDEINI Ortmann, 1916**								

Sinosolenaia carinata (Heude, 1877)	n/a	China	n/a	n/a	KX822669	NC_023250	KX822626	
Gonidea angulata (Lea, 1838)	RMBH biv0294_1	USA: Okanagan Lake	n/a	n/a	MN402615	MN396726	MN396722	
Leguminaia wheatleyi (Lea, 1862)	RMBH biv0177_7	Turkey: Karasu River	n/a	n/a	MN402614	MN396725	MN396721	
**Tribe LAMPROTULINI Modell, 1942**								
Lamprotula leaii (Griffith & Pidgeon, 1833)	RMBH biv0200_1	Vietnam	n/a	n/a	MN402616	MN396727	MN396723	
Potomida littoralis (Cuvier, 1798)	RMBH biv0177_10	Turkey: Karasu River	n/a	n/a	MN402617	MN396728	MN396724	
Pronodularia japanensis (Lea, 1859)	NCSM 27183	Japan	n/a	n/a	KX822659	AB055625	KX822615	
**Tribe CHAMBERLAININI Bogan, Froufe & Lopes-Lima in Lopes-Lima et al. 2017**								
Chamberlainia hainesiana (Lea, 1856)	BIV46	Thailand	n/a	n/a	NC_044110	n/a	KX822592	
Sinohyriopsis cumingii (Lea, 1852)	16_NCU_XPWU_SU177	China	n/a	n/a	NC_011763	NC_011763	MG595614	
**Tribe RECTIDENTINI Modell, 1942**								
Hyriopsis bialata Simpson, 1900	BIV1774	Malaysia: Pahang, Pahang River	n/a	n/a	KX051274	MT993644	MT993697	
Ensidens ingallsianus (Lea, 1852)	MUMNH-UNI0074	Thailand: Chao Phraya River Basin, Phrae, Song River	n/a	n/a	MT993541	MT993687	MT993739	
Rectidens sumatrensis (Dunker, 1852)	RMBH biv0211_1	Malaysia: Perak River, Perak	n/a	n/a	MF352288	n/a	MF352405	
**Tribe CONTRADENTINI Modell, 1942**								
Lens contradens (Lea, 1838)	MUMNH-UNI0197	Thailand: Chao Phraya River Basin, Uthai Thani, Sakae Krang River	n/a	n/a	MG581991	MT993693	MT993745	
Physunio superbus (Lea, 1843)	MUMNH-UNI0199	Thailand: Chao Phraya River Basin, Nakhon Sawan, Chao Phraya River,	n/a	n/a	MG582020	MT993689	MT993741	
Trapezoideus foliaceus (Gould, 1843)	UF507879 (=ICH-02104)	Thailand: Mae Klong River basin, Pachee River	n/a	n/a	MH345984	MH346024	MH346004	
**Subfamily UNIONINAE Rafinesque, 1820**								
**Tribe UNIONINI Rafinesque, 1820**								
Unio crassus Philipsson in Retzius, 1788	n/a	France	n/a	n/a	KC703878	n/a	KC703644	
Unio pictorum (Linnaeus, 1758)	n/a	Europe	n/a	n/a	KC429109	n/a	KC429447	
Unio tumidus Philipsson in Retzius, 1788	n/a	Ukraine	n/a	n/a	KX822672	n/a	KX822630	
Aculamprotula tortuosa (Lea, 1865)	n/a	China	n/a	n/a	KX822631	n/a	KX822586	
Arcuneopsis pisciculus (Heude, 1874)	n/a	China	n/a	n/a	KX822639	n/a	KX822596	
Cuneopsis heudei (Heude, 1874)	n/a	China	n/a	n/a	KX822638	n/a	KX822595	
Cuneopsis rufescens (Heude, 1874)	n/a	China	n/a	n/a	KX822640	n/a	KX822597	
Nodularia douglasiae (Griffith & Pidgeon, 1833)	n/a	China	n/a	n/a	KX822653	n/a	KX822610	
Nodularia nuxpersicae (Dunker, 1848)	NCSM84990	Vietnam	n/a	n/a	KX822654	n/a	KX822611	
Schistodesmus lampreyanus (Baird & Adams, 1867)	n/a	China	n/a	n/a	KX822665	n/a	KX822621	
**Tribe ANODONTINI Rafinesque, 1820**								
**Subtribe ALASMIDONTINA Rafinesque, 1820**								
Alasmidonta marginata Say, 1818	UMMZ265695	USA	n/a	n/a	AF156502	n/a	AF400688	
Lasmigona compressa (Lea, 1829)	UMMZ265696	USA	n/a	n/a	AF156503	n/a	DQ191414	
Pyganodon grandis (Say, 1829)	n/a	USA	n/a	n/a	AF231734	n/a	AF305384	

Strophitus undulatus (Say, 1817)	UMMZ265693	USA	n/a	n/a	AF156505	n/a	DQ191415	
Simpsonaias ambigua (Say, 1825)	NCSM30607	USA	n/a	n/a	KX822666	n/a	KX822622	
**Subtribe ANODONTINA Rafinesque, 1820**								
Anodonta anatina (Linnaeus, 1758)	EU	European	n/a	n/a	KX822632	n/a	KX822588	
Anodonta cygnea (Linnaeus, 1758)	n/a	Italy	n/a	n/a	KX822633	n/a	KX822589	
Anodonta nuttalliana Lea, 1838	n/a	USA	n/a	n/a	KX822634	n/a	KX822590	
Pseudanodonta complanata (Rossmässler, 1835)	hap247	Ukraine	n/a	n/a	KX822661	n/a	KX822617	
**Subtribe CRISTARIINA Lopes-Lima, Bogan & Froufe in Lopes-Lima et al., 2017**								
Cristaria plicata (Leach, 1814)	n/a	Vietnam	n/a	n/a	KY561634	n/a	KY561666	
Sinanodonta lucida (Heude, 1877)	n/a	China	n/a	n/a	KX822667	n/a	KX822624	
Sinanodonta jourdyi (Morlet, 1886)	n/a	Vietnam	n/a	n/a	KY561635	KY561649	KY561667	
Sinanodonta lauta (Martens, 1877)	n/a	Siberia	n/a	n/a	KY561633	KY561648	KY561665	
**Tribe LANCEOLARIINI Froufe, Lopes-Lima, & Bogan in Lopes-Lima et al., 2017**								
Lanceolaria gladiola (Heude, 1877)	n/a	China	n/a	n/a	KX822648	n/a	KX822605	
Lanceolaria grayii (Griffith & Pidgeon, 1833)	n/a	China	n/a	n/a	KX822649	n/a	KX822606	
Lanceolaria grayii (Griffith & Pidgeon, 1833)	n/a	Vietnam	n/a	n/a	KX822650	n/a	KX822607	
Pletholophus tenuis (Griffith & Pidgeon, 1833)	NCSM84924	Vietnam	n/a	n/a	KX822658	n/a	KX822614	
**Subfamily AMBLEMINAE Rafinesque, 1820**								
**Tribe AMBLEMINI Rafinesque, 1820**								
Amblema plicata (Say, 1817)	n/a	USA	n/a	n/a	U56841	n/a	AF305385	
**Tribe LAMPSILINI Ihering, 1901**								
Lampsilis cardium Rafinesque,1820	n/a	USA	n/a	n/a	AF120653	n/a	AF305386	
Actinonaias ligamentina (Lamarck,1819)	n/a	USA	n/a	n/a	AF156517	n/a	DQ191420	
Villosa iris (Lea,1829)	n/a	USA	n/a	n/a	AF156524	n/a	DQ191422	
**Tribe PLEUROBEMINI Hannibal, 1912**								
Elliptio complanata (Lightfoot, 1786)	n/a	USA	n/a	n/a	EU448173	n/a	JF899181	
Elliptio dilatata (Rafinesque, 1820)	n/a	USA	n/a	n/a	AF156507	n/a	AF400690	
Pleurobema sintoxia (Rafinesque, 1820)	n/a	USA	n/a	n/a	AF156509	n/a	DQ191418	
**Tribe QUADRULINI Ihering, 1901**								
Quadrula quadrula (Rafinesque, 1820)	n/a	USA	n/a	n/a	AF156511	n/a	DQ191417	
Quadrula verrucosa (Rafinesque, 1820)	n/a	USA	n/a	n/a	DQ191413	n/a	DQ191416	
**Family MARGARITIFERIDAE Henderson, 1929**								
**subfamily MARGARITIFERINAE Henderson, 1929**								
Margaritifera dahurica (Middendorff, 1850)	biv 92_6	Far East of Russia: Amur River basin, Ilistaya River	n/a	n/a	KJ161516	KJ943526	KT343747	
Margaritifera margaritifera (Linnaeus, 1758)	n/a	Northwestern Russia: Onega River basin, Somba River	n/a	n/a	KX550089	KX550091	KX550093	
Margaritifera laevis (Haas, 1910)	biv d0036/22	Far East of Russia: Kurile Archipelago, Kunashir Island, Sennaya River	n/a	n/a	KJ161500	KJ943523	KT343742	

Margaritifera middendorffi (Rosén, 1926)	biv d0099/6	Far East of Russia: Russia: Kamchatka, Bolshaya River basin, Nachilova River	n/a	n/a	KJ161547	KJ943528	KT343745	
Margaritifera falcata (Gould, 1850)	n/a	USA: Idaho, Fremont Co., Buffalo River	n/a	n/a	AY579128	AY579085	n/a	
Margaritifera marrianae Johnson, 1983	n/a	USA	n/a	n/a	HM849098	AY579086	n/a	
Pseudunio auricularius (Spengler, 1793)	n/a	Spain: Tarragona, Ebro River	n/a	n/a	AY579125	AY579083	n/a	
Pseudunio marocanus (Pallary, 1918)	n/a	Morocco: Oum Er-Rbia River basin, ElAbid River	n/a	n/a	EU429679	EU429691	n/a	
Cumberlandia monodonta (Say, 1829)	Hap028	USA: Mississippi River basin	n/a	n/a	AY579131	AY579089	AF305382	
**Subfamily GIBBOSULINAE Bogan, Bolotov, Froufe & Lopes-Lima in Lopes-Lima et al., 2018**								
Gibbosula laosensis (Lea, 1863)	biv186_1	Laos: Mekong River basin, Nam Long River		n/a	JX497731	KC845943	KT343741	

Supplementary Table S2 The most probable ancestral areas with the inferred biogeographic events and the mean age of the most recent common ancestor with 95% highest posterior density (HPD) of the primary clades of *Pilsbryoconcha* in Indochina. Results of ancestral area reconstruction were inreferred from three probabilistic algorithms: Statistical Dispersal-Vicariance Analysis (S-DIVA), Statistical Dispersal-Extinction-Cladogenesis (S-DEC), and Bayesian binary MCMC analysis (BBM), and the combined results from the three analyses, as implemented in RASP 4.2. The distribution areas (see map in fig.2) are Chao Phraya Basin (A), Sakon Nakhon Basin (F), Khorat Plateau Basin (G), and Mekong Delta (H). Mean age and 95% HPD were calculated using fossil calibration as implemented in BEAST v.2.6.1. Time scale in million years ago (Mya).

Clade	Biogeographic event	The most probable ancestral areas				Mean age (Mya)	95% HPD (Mya)
		S-DIVA	S-DEC	BBM	Combined results		
Clade I	Vicariance	A+G (47%)	G (42%)	A (64%)	A+G (30%)	18.11	10.84–26.63
P. exilis	Intra-area radiation	A (55%)	A+G (92%)	A (98%)	A (53%)	4.11	2.42–6.06
P. schomburgki	Dispersal	G (81%)	B+G (55%)	G (53%)	G (48%)	2.61	1.17–4.24
Clade II	Vicariance	F+H (76%)	F+H (18%)	F (60%)	F +H (29%)	24.02	16.59–32.11
Clade III	Dispersal	H (99%)	A+H (60%)	H (91%)	H (73%)	9.44	5.01–14.53
P. carinifera	Intra-area radiation	H (100%)	H (50%)	H (99%)	H (83%)	1.91	0.83–3.26
P. linguaeformis	Vicariance	A+H (100%)	A+H (98%)	H (83%)	A+H (67%)	3.50	1.36–6.22
Clade IV	Dispersal	F (72%)	F+G (56%)	F (96%)	F (66%)	17.71	11.90–24.21
P. acuta	Intra-area radiation	G (100%)	G (100%)	G (98%)	G (99%)	5.32	3.00–7.97
P. kittitati	Intra-area radiation	G (100%)	G (100%)	G (99%)	G (99%)	0.76	0.10–1.71
P. mekongiana	Intra-area radiation	F (100%)	F (100%)	F (99%)	F (99%)	1.71	0.63–3.03
P. hoikaab	Intra-area radiation	F (100%)	F (100%)	F (99%)	F (100%)	2.41	1.15–3.89
Pilsbryoconcha	Dispersal/Vicariance	F+G (38%)	A+G (21%)	F (44%)	F+G (19%)	30.20	21.22–49.86
Pilsbryoconcha + *Namkongnaia* + *Monodontina*	Dispersal	F (69%)	F (21%)	F (50%)	F (46%)	43.12	31.14–56.27

Supplementary Table S3 Shell measurements for the type series of new species. Measurements in millimeters (mm).

Taxa	Type status	Specimen Voucher (MUMNH)	Shell length	Shell height	Shell width	height/length
Pilsbryoconcha mekongiana Jeratthitikul & Prasankok sp. nov.	Holotype	UNI0843	106.18	52.49	18.84	0.49
	Paratype	UNI0840	109.24	54.43	20.22	0.50
	Paratype	UNI0841	114.65	53.36	18.96	0.47
	Paratype	UNI0842	106.91	49.77	17.87	0.47
	Paratype	UNI0844	100.18	48.08	16.17	0.48
Pilsbryoconcha hoikaab Jeratthitikul & Prasankok sp. nov.	Holotype	UNI0305	92.32	47.27	15.32	0.51
	Paratype	UNI0306	95.51	48.69	17.01	0.51
Pilsbryoconcha acuta Jeratthitikul & Prasankok sp. nov.	Holotype	UNI1510	83.59	39.13	16.07	0.47
	Paratype	UNI1509	74.05	37.16	14.54	0.50
Pilsbryoconcha kittitati Jeratthitikul & Prasankok sp. nov.	Holotype	UNI0372	90.69	40.42	21.32	0.45
	Paratype	UNI0374	77.94	37.97	17.08	0.49

Supplementary Data S1 Other material examined

Genus *Pilsbryoconcha* Simpson, 1900

***Pilsbryoconcha exilis* (Lea, 1838)**

Materials examined: **INDONESIA** • 3 shells; Bogor Botanical Gardens, Bogor City, West Java; 6°35'58.4"S, 106°48'09.7"E; MUMNH-UNI2481 to UNI2483. **THAILAND** • 1 shell; Ping River at Nong Long, Wiang Nong Long District, Lamphun Province; 18°25'30.2"N, 98°42'4.4"E; MUMNH-UNI0312 • 3 shells; Song River at Ban Klang, Song District, Phrae Province; 18°27'47.2"N, 100°11'01.8"E; MUMNH-UNI0070, UNI0071, and UNI 2773 • 3 shells; Mae Lai River at Ban Mae Lai, Mueang District, Phrae Province; 18°13'11.0"N, 100°12'24.0"E; MUMNH-UNI0086 to UNI0088 • 1 shell; Sakae Krang River at Nong Phai Baen, Mueang District, Uthai Thani Province; 15°25'43.6"N, 100°03'10.9"E; MUMNH-UNI2779 • 1 shell; Pasak River at Sapradu, Wichian Buri District, Phetchabun Province; 15°38'56.4"N, 101°06'01.3"E; MUMNH-UNI0401 • 1 shell; Unnaned Pond at Sak Long, Lom Sak District, Phetchabun Province; 16°49'18.3"N, 101°15'43.7"E; MUMNH-UNI0135 • 2 shells; Bueng Tabaek, Nong Chaeng, Bueng Sam Phan District, Phetchabun Province; 15°49'58.7"N, 101°02'05.3"E; MUMNH-UNI0594 and UNI0595 • 2 shells; Pasak River at Sapradu, Wichian Buri District, Phetchabun Province; 15°38'56.4"N, 101°06'01.3"E; MUMNH-UNI0678 and UNI0679 • 1 shell; Pasak River at Nayom, Mueang District, Phetchabun Province; 16°15'02.2"N, 101°07'36.4"E; MUMNH-UNI0290 • 3 shells; Sablek Reservoir, Kok Toom, Mueang District, Lop Buri Province; 14°49'37.5"N, 100°47'35.5"E; MUMNH-UNI2702, UNI2774, and UNI 2775 • 3 shells; Nong Tapong Pond, Makham District, Chanthaburi Province; 12°40'43.0"N, 102°12'10.3"E; MUMNH-UNI2776 to UNI2778 • 4 shells; Sai Khao Stream, Soi Dao District, Chanthaburi Province; 13°04'20.8"N, 102°19'19.4"E; MUMNH-UNI0751 to UNI0754 • 5 shells; Mahidol University, Salaya Campus, Phutthamonthon District, Nakhon Pathom Province; 13°47'33.5"N, 100°19'16.7"E; MUMNH-UNI2780 to UNI2784 • 12 shells; Mueang Canal at Si Chula Temple, Mueang District, Nakhon Nayok Province; 14°05'39.7"N, 101°10'17.8"E; MUMNH-UNI2788 to UNI2791 • 5 shells; Unnamed stream near Klong Hat, Khlong Hat District, Sa Kaeo Province; 13°27'39.5"N, 102°15'46.7"E; MUMNH-UNI2792 to UNI2796 • 6 shells; Unnamed stream near Klong Hinpun, Wang Nam Yen District, Sa Kaeo Province; 13°37'18.0"N, 102°06'35.1"E; MUMNH-UNI0417 to UNI0421, and UNI2772 • 2 shells; Unnamed pond near Sam Phrao, Mueang District, Udon Thani; 17°27'15.9"N, 102°52'37.8"E; MUMNH-UNI0662, and UNI0663 • 1 shell; Unnamed pond near Nong Ya Sai, Wang Sam Mo District, Udon Thani Province; 16°58'47.4"N, 103°20'13.4"E; MUMNH-UNI0373 • 5 shells; Thepsuda Bridge, Lam Pao Dam, Sahatsakhan District, Kalasin Province, 16°42'54.7"N, 103°27'58.0"E; MUMNH-UNI1517 to UNI1521 • 1 shell; Chi River, Changhan District, Roi Et Province; 16°12'10.2"N, 103°33'23.7"E; MUMNH-UNI2701 • 3 shells; Lam Munbon Dam, Khon Buri District, Nakhon Ratchasima Province; 14°28'27.9"N, 102°07'25.9"E; MUMNH-UNI2785 to UNI2789 • 1 shell; Unnamed Pond, Warin Chamrap District, Ubon Ratchathani Province; 15°08'20.5"N, 104°51'59.4"E; MUMNH-UNI2700.

***Pilsbryoconcha schomburgki* (Martens, 1860) stat. rev.**

Other materials examined: **THAILAND** • 2 shells; Khlong Taphoen Stream at Lat Ya, Mueang District, Kanchanaburi Province, 14°08'13.8"N, 99°22'59.6"E; MUMNH-UNI1515 and UNI1516 • 1 shell; Mae Klong River at Tha Pha, Ban Pong District, Ratchaburi Province; 13°51'28.7"N, 99°49'19.1"E; MUMNH-UNI1514 • 5 shells; Mae Klong River at Nong Klang Na, Mueang District, Ratchaburi Province; 13°34'34.4"N, 99°48'59.7"E; MUMNH-UNI0581 to UNI0585 • 3 shells; Nong Ta Khong Pond, Mueang District, Nakhon Ratchasima Province; 14°56'38.3"N, 102°09'38.0"E; MUMNH-UNI2797 to UNI2799 • 5 shells; Unnamed Stream at Ban Lum Khao, Pak Thong Chai District, Nakhon Ratchasima; 14°41'07.8"N, 102°03'55.4"E; MUMNH-UNI2800 to UNI2804 • 2 shells; Sabpradu Reservoir, Sikhio District, Nakhon Ratchasima Province; 14°50'20.1"N, 101°41'06.8"E; MUMNH-UNI2805 and UNI2806 • 7 shells; Khlong Phraphut Stream at Nong Ta Khong, Pong

Nam Ron District, Chanthaburi; 13°02'39.2"N, 102°25'05.1"E; MUMNH-UNI1522 to UNI1528 • 3 shells; Lam Samlai Reservoir, Pak Thong Chai District, Nakhon Ratchasima Province; 14°40'09.1"N, 101°52'05.4"E; MUMNH-UNI2807 to UNI2809 • 3 shells; Lam Phra Phloeng Stream at Wangmi, Wang Nam Khiao District, Nakhon Ratchasima Province; 14°27'43.5"N, 101°39'38.0"E; MUMNH-UNI2810 to UNI2812 • 1 shell; Khlong Min Stream at Chandi, Chawang District, Nakhon Si Thammarat Province; 8°22'52.5"N, 99°31'22.3"E; MUMNH-UNI0836.

Pilsbryoconcha carinifera **(Conrad, 1837)**

Other materials examined: THAILAND • 1 shell; Mueang Canal at Si Chula Temple, Mueang District, Nakhon Nayok Province; 14°05'39.7"N, 101°10'17.8"E; MUMNH-UNI2813 • 1 shell; Huai Yang Stream at Sae O, Watthana Nakhon District, Sa Kaeo Provinc; 13°55'31.5"N, 102°31'37.7"E; MUMNH-UNI2818 to UNI2822 • 2 shells; Huai Yang Stream at Thap Sadet, Ta Phraya District, Sa Kaeo Province; 14°02'00.4"N, 102°49'35.4"E; MUMNH-UNI2814 and UNI2815 • 2 shells; Khlong Phrom Hot Stream at Phak Kha, Watthana Nakhon District, Sa Kaeo Province; 13°44'56.6"N, 102°25'37.6"E; MUMNH-UNI2816 and UNI2817. **CAMBODIA** • 4 shells; Paoy Samraong, Preah Netr Preah, Preah Netr Preah District, Banteay Meanchey Province; 13°35'01.8"N, 103°09'24.8"E; MUMNH-UNI2645, UNI2736, UNI2737 and UNI2771.

Pilsbryoconcha linguaeformis **(Morelet, 1875)**

Other materials examined: CAMBODIA • 3 shells; Don Sdeung, Boeung Chhmar Fish Sanctuary, Staung District, Kampong Thom Province; 12°47'25.1"N, 104°17'55.7"E; MUMNH-UNI2625, UNI2738 and UNI2739 • 1 shell; Kampong Chhnok Tru landing point, Boribo District, Kampong Chhnang Province; 12°30'36.5"N, 104°27' 18.2"E; MUMNH-UNI2624 • 3 shells; Tonle Sap Lake open area near Chong Khneas, Chong Khneas, Siem Reap Province; 12°30'20.1"N, 103°50'06.2"E; MUMNH-UNI2742 to UNI2744 • 1 shell; Tonle Sap River in Kampong Prasat, Saeb, Kampong Tralach District, Kampong Chhnang Province; 12°04'23.6"N, 104°46'23.8"E; MUMNH-UNI2619 • 3 shells; Tonle Sap Lake open area, Krakor District, Pursat Province; 12°35'42.1"N, 104°12'13.1"E; MUMNH-UNI2636, UNI2740, and UNI2741. **THAILAND** • 1 shell; Huai Yang Stream at Sam Phan Ta, Na Di District, Prachin Buri Province; 14°07'44.8"N, 101°44'50.3"E; MUMNH-UNI2698.

Pilsbryoconcha hoikaab **Jeratthitikul & Prasankok sp. nov.**

Other materials examined: THAILAND • 3 shells; Songkhram River at Thung Fon, Thung Fon District, Udon Thani Province; 17°27'06.5"N, 103°16'50.4"E; MUMNH-UNI0821 to UNI0823 • 4 shells; Songkhram River at Ban Ya, Nong Han District, Udon Thani Province; 17°23'44.5"N, 103°18'01.7"E; MUMNH-UNI0316 to UNI0319 • 3 shells; Songkhram River at Tha Sa-at, Seka District, Bueng Kan Province; 17°55'54.7"N, 103°45'36.9"E; MUMNH-UNI0673 to 0675 • 1 shell; Songkhram River at Chai Buri, Tha Uthen District, Nakhon Phanom Province; 17°38'59.8"N, 104°27'45.6"E; MUMNH-UNI0186 • 1 shell; That Phanom District, Nakhon Phanom Province; 16°58'46.6"N, 104°43'50.4"E; MUMNH-UNI2705 • 5 shells; Yam Stream at Kae Dam, Charoen Sin District, Sakon Nakhon Province; 17°31'56.9"N, 103°33'39.5"E; MUMNH-UNI0351 to 0355. **LAOS** • 1 shell; Nam Hin Boun River at Hin Boun City, Khammouane Province; 17°35'46.8"N 104°37'04.9"E; MUMNH-UNI2710 • 3 shells; Nam Pa Kan River at Mai Nam Pakan, Khammouane Province; 17°39'52.2"N, 104°37'37.0"E; MUMNH-UNI2719 to UNI2721 • 2 shells; Nam Don River at Thakhek, Khammouane Province; 17°26'32.9"N, 104°51'22.2"E; MUMNH-UNI2708 and UNI2709 • 8 shells; Xe Nou River at Ban Kèngmun, Savannakhet Province; 17°4'22.34"N, 105°2'54.97"E; MUMNH-UNI2711 to UNI2718.

Pilsbryoconcha mekongiana **Jeratthitikul & Prasankok sp. nov.**

Other materials examined: **THAILAND** • 5 shells; Unnamed Pond near Sam Phrao, Mueang District, Udon Thani Province; 17°27'15.9"N, 102°52'37.8"E; MUMNH-UNI0660 to UNI0666• 5 shells; Huai Nam Suai Stream at Khok Chang, Sakhrai District, Udon Thani Province; 17°39'50.7"N, 102°46'22.3"E; MUMNH-UNI2547 to UNI2551.

Pilsbryoconcha acuta **Jeratthitikul & Prasankok sp. nov.**

Other materials examined: **THAILAND** • 3 shells; Lam Phra Phloeng Stream at Nok Ok, Pak Thong Chai District, Nakhon Ratchasima Province; 14°41'34.9"N, 102°03'18.0"E; MUMNH-UNI1529 to UNI1531 • 1 shell; Mun River at Krabueang, Chumphon Buri District, Surin Province; 15°18'55.1"N, 103°17'12.2"E; MUMNH-UNI2703 • 1 shell; Unnamed Stream on Chok Chai-Det Udom Road, Surin Province; 14°37'11.0"N, 103°37'37.4"E; MUMNH-UNI2704 • 1 shell; Mun River at Ban Tha Tum, Tha Tum District, Surin Province; MUMNH-UNI2724 • 1 shell; Tributary of Mun River, Khong Chiam District, Ubon Ratchathani Province; 15°18'26.1"N, 105°29'44.9"E; MUMNH-UNI1513 • 3 shells; Unnamed stream at Na Krasaeng, Det Udom District, Ubon Ratchathani Province; 14°51'11.8"N, 104°56'02.8"E; MUMNH-UNI2725 to UNI2727. **LAOS** • 2 shells; Unnamed Pond at Pakse, Champasak Province; 15°07'48.7"N, 105°43'44.3"E; MUMNH-UNI2722 and UNI2723.
